# Structural and Mechanical Durability Evaluation of PLLA Scaffolds for Biomedical Applications

**DOI:** 10.3390/ma19173759

**Published:** 2026-09-04

**Authors:** Jagoda Kurowiak, Aleksandra Łysiak, Tomasz Klekiel

**Affiliations:** Department of Biomedical Engineering, Institute of Material and Biomedical Engineering, Faculty of Engineering and Technology, University of Zielona Gora, Licealna 9 Street, 65-417 Zielona Gora, Poland; a.lysiak03@gmail.com (A.Ł.); tklekiel@uz.zgora.pl (T.K.)

**Keywords:** poly(L-lactide), scaffold, tissue engineering, regenerative medicines, hydrolytic degradation, viscoelasticity, constitutive modeling

## Abstract

Poly(L-lactide) (PLLA) scaffolds are widely used in tissue engineering because they are biodegradable and have good mechanical properties. Their performance changes during degradation, which is important for the design of biodegradable implants. In this study, we investigated the structural and mechanical stability of 3D-printed PLLA scaffolds during 180 days of in vitro hydrolytic degradation in phosphate-buffered saline (PBS) at 37 °C. Mechanical properties of the material were evaluated by tensile and compression tests. The material during degradation was analyzed for the scaffolds for which the lost mass and chemical and surface changes were analyzed using FTIR-ATR spectroscopy and SEM microscopy. The scaffolds lost more than 8% of their initial mass throughout the study, indicating slow degradation. However, their mechanical properties gradually decreased. Young’s modulus dropped from 2.46 GPa to 2.09 GPa, and tensile strength decreased from 45.5 MPa to 31.1 MPa. The material also became more brittle after about 90 days. FTIR results confirmed progressive hydrolysis of ester bonds, while SEM images showed increasing surface roughness, micropores, and cracks. The results show that PLLA scaffolds maintain their shape and mass during the early stages of degradation. However, their mechanical strength decreases over time. These changes should be considered when designing biodegradable scaffolds for regenerative medicine.

## 1. Introduction

Tissue engineering is an interdisciplinary field of science that combines knowledge from biology, medicine, and engineering. It focuses on developing innovative therapeutic approaches. The term “tissue engineering” was officially coined in 1988 during a National Science Foundation workshop [1,2]. Progress in the development of tissue engineering indicates that it may serve as a promising alternative to conventional therapies.

The human body is composed of tissues and organized structures across various scales (from micro to nano) designed to fulfill specific functions [3]. The development of novel, multifunctional materials represents an ideal solution for the repair and regeneration of damaged tissues. This is of paramount importance for medicine. Unfortunately, the number of patients suffering from total or partial tissue and organ damage is increasing year by year. Traditional treatment methods are no longer sufficient, and the challenges in acquiring potential donors continue to grow. In response to these issues, the field of tissue engineering has emerged within biomedicine [3,4].

Biomaterials used in biomedicine play a crucial role in the regeneration process of damaged tissues, frequently integrating with them permanently. They interact directly with the biological system without disrupting host homeostasis; therefore, they must satisfy a range of rigorous requirements. Biomaterial-based scaffolds should exhibit biocompatibility, meaning they must not induce inflammatory, oncogenic, or mutagenic responses. Furthermore, bioresorbability and appropriate mechanical properties—such as elasticity, compressive and tensile strength, and stiffness—are of fundamental importance. Additionally, biomaterials must promote cellular interactions, including cell adhesion, proliferation, migration, and differentiation capacity [5,6]. The material structure should feature high porosity to facilitate proper tissue development. Biodegradability represents another key aspect. The degradation rate of the biomaterial must be tailored and sufficient to match the time required for new tissue formation, while the degradation byproducts must not be harmful to the organism. These materials must be non-toxic, free of hazardous substance release during decomposition, and manufactured in a reproducible manner [7].

Polymers are extensively investigated for their promising roles in various biomedical fields, such as drug delivery, biosensors, and tissue regeneration [8,9]. Owing to their unique mechanical and physicochemical properties, their range of applications is exceptionally broad [10]. The selection of polymeric materials varies depending on the specific site of application. The dynamic advancement of materials engineering enables the design of polymers with tailored biocompatibility, porosity, and mechanical strength. Given that polymers constitute a vast class of materials, they can be categorized into those of natural and synthetic origin [11]. Biodegradable polymers, such as lactic acid derivatives, enable the fabrication of structures that support bone tissue regeneration and the angiogenesis process, while simultaneously undergoing controlled degradation. Synthetic biopolymers can be successfully utilized as scaffolds for damaged tissues. Among these, poly(L-lactide) (PLLA) stands out, being highly valued for the ability to modulate its mechanical parameters and biodegradation rate. Furthermore, this material allows for the fabrication of porous scaffolds with diverse micro- and nanostructures using various processing technologies [12].

Poly(L-lactide) (PLLA) is an aliphatic polyester derived from L-lactic acid, a chiral organic molecule with an L-configuration obtained from plant sources [13]. The chirality of lactic acid leads to the existence of several polylactide forms: PLLA, PDLA (poly(D-lactic acid)), and PDLLA, which is a mixture of D- and L-isomers [14,15]. Among these forms, PLLA exhibits the highest stereoregularity and crystallinity, resulting from the exclusive presence of L-lactic units within the macromolecular chain [16]. The ester bonds constituting the polymer backbone are responsible for its susceptibility to hydrolysis, which leads to the gradual degradation of the material into lactic acid, subsequently metabolized by the human body [14].

The production of PLLA most commonly proceeds via the ring-opening polymerization of the cyclic dimer of lactic acid [15]. Only L-lactide is utilized for PLLA synthesis, as maintaining a uniform stereoisomeric configuration is crucial for achieving high crystallinity and a large molecular weight [15,17]. The stereoregularity of the PLLA molecular chain allows this polymer to achieve a crystallinity in the range of 35–40%, which significantly distinguishes it from PDLLA, which is an amorphous material [14]. High crystallinity results from the ordered arrangement of methyl groups along the chain, which restricts its mobility and promotes the formation of spherulites—supramolecular structures typical for semicrystalline materials [16]. At the macrostructural level, the presence of these ordered regions ensures that PLLA is characterized by greater stiffness, thermal stability, and a slower degradation rate compared to other polymers [14]. These features render PLLA one of the most frequently selected polymers for biomedical applications, particularly where mechanical stability and controlled degradation are essential [13,18].

PLLA is used as a component of copolymers in tissue engineering scaffolds due to its slow degradation. It is a synthetic material characterized by good biocompatibility, hemocompatibility, and favorable mechanical properties [19]. The mechanical properties of bioresorbable PLLA-based scaffolds feature a tensile strength within the range of 60–70 MPa, an elastic modulus ranging from 2 to 4 GPa, and an elongation at break of 2–6% [17,20]. The properties of PLLA depend on several factors, including molecular weight, degree of crystallinity, and changes occurring within the polymer chain as the material ages [21]. Due to the feasibility of modifying its properties, it has been adapted for the fabrication of scaffolds designed for diverse applications, such as the regeneration of bone, cartilage, skin, and cardiovascular tissues.

Bone tissue is a dynamic structure in which various cell types (osteoblasts, osteoclasts, and osteogenic cells) are responsible for maintaining tissue balance [22,23,24]. Minor bone injuries heal spontaneously, but extensive or complex defects still pose a serious therapeutic challenge [25,26]. In such cases, PLLA scaffolds can provide significant support for regeneration. In vitro studies have shown that PLLA scaffolds modified with various additives, such as hydroxyapatite (HA), promote cell growth and collagen production [27].

On the other hand, in vivo studies confirmed that PLLA and PLLA/PCL scaffolds can support bone regeneration, with the PLLA/PCL composite exhibiting enhanced callus formation, partly because PCL does not generate acidic degradation byproducts [28]. Other experimental studies have shown that, in a rat skull defect model, PLLA/gelatin and PLLA/gelatin/HA scaffolds exhibited beneficial regenerative properties, although the differences between them disappeared after 10 weeks [29]. Additionally, additive manufacturing technologies, including low-temperature deposition (LDM, Liquid Deposition Modeling), have enabled the creation of PLLA composites with bioceramic materials, resulting in highly porous scaffolds with strength comparable to cancellous bone and good bone conduction in vivo [30,31].

Cartilage is a connective tissue in which chondrocytes are surrounded by a matrix rich in water, collagen, and proteoglycans [32]. Its ability to self-repair is very limited; therefore, damage caused by injury or aging often leads to diseases such as osteoarthritis [33]. For this reason, tissue engineering is developing various types of scaffolds, sources of mesenchymal stem cells, and combinations of synthetic polymers with cells to create effective implants for cartilage regeneration [34]. For example, Rajzer et al. [35] developed layered gelatin/PLLA scaffolds by combining electrospinning and 3D printing, achieving tensile strength comparable to that of nasal cartilage. Mallick et al. [36] created hybrid chitosan/PLLA scaffolds that exhibited favorable mechanical and cellular properties. Conoscenti et al. [37], in turn, demonstrated that PLLA scaffolds with pore sizes of approximately 100 µm better support the expression of chondrogenic genes than structures with larger pores, for both articular chondrocytes and nasal septum chondrocytes. Appropriate porosity of PLLA scaffolds significantly improves their functionality, making them a promising platform for cartilage regeneration.

In the treatment of myocardial infarction caused by atherosclerosis, coronary artery bypass grafting is most commonly used; however, this procedure merely bypasses the damaged vessels without regenerating heart tissue [38]. Therefore, tissue engineering aims to create scaffolds that replicate the structure and function of native blood vessels. Arteries have a three-layered structure, which makes developing an artificial counterpart a major challenge [19]. A new approach proposed by Javanmard et al. [39] involves multilayer scaffolds produced by sequential electrospinning. The use of a combination of PCL, collagen, and PLLA made it possible to replicate the three layers of an artery, and studies have shown good adhesion and growth of both endothelial and smooth muscle cells, with no signs of cytotoxicity.

The primary objective of the presented study was to analyse the structural and mechanical stability of poly(L-lactide) (PLLA) scaffolds intended for biomedical applications, specifically focusing on how their properties evolve during an in vitro hydrolytic degradation process. The authors sought to determine whether chemical changes in the polymer matrix were reflected in the scaffold’s mechanical performance and structural morphology throughout the 180-day degradation period.

The research methodology was based on a multi-parametric experimental approach. PLLA scaffolds were fabricated using Fused Deposition Modelling (FDM) 3D printing. The methodology for evaluation included:

- Gravimetric analysis to measure mass loss;
- Static tensile and compression testing to determine Young’s modulus and yield strength;
- Fourier-Transform Infrared Spectroscopy (FTIR) using the Integrated Difference Spectrum (IDS) method to quantify chemical changes;
- Field Emission Scanning Electron Microscopy (SEM) to observe surface morphology.

The specimens were divided into two geometries: tensile test samples (standardized to ISO 527) and cylindrical scaffolds for compression tests. Degradation was simulated via incubation in Phosphate-Buffered Saline (PBS) at 37 °C for 180 days.

## 2. Materials and Methods

### 2.1. Materials

For the experimental investigations, a poly(L-lactic acid) (PLLA) filament from 3D4MAKERS (Haarlem, The Netherlands) with a nominal diameter of 1.75 mm was utilized. The density of the material was 1.24 g/cm^3^, and its Shore hardness was determined to be 81 (D scale). Phosphate-buffered saline (PBS) solution with a pH of 7.4 was employed as the degradation medium. The PBS solution was prepared according to the formulation provided by Vedeanu et al. [40], comprising: NaCl 8 g/L, KCl 0.2 g/L, Na_2_HPO_4_ 1.14 g/L and KH_2_PO_4_ 0.245 g/L. All analytical-grade chemical reagents were purchased from Sigma-Aldrich (St. Louis, MO, USA). Deionized water was used for the preparation of all solutions.

### 2.2. Scaffold Design and Fabrication

Two types of samples were prepared using the fused deposition modeling (FDM) method. The 3D printing process was carried out using a Flashforge Adventurer 3 printer (Zhejiang Flashforge 3D Technology Co., Ltd., Jinhua, China). In accordance with the adopted research methodology, the components had strictly defined geometries.

**Figure 1:**
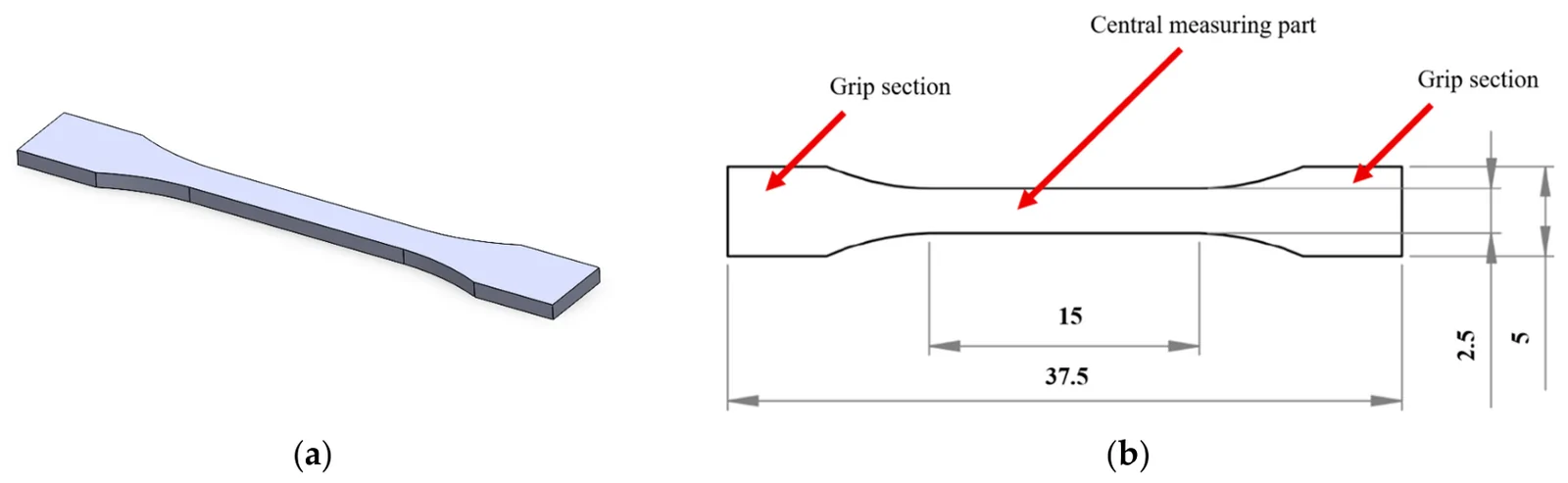
PLLA specimen 3D model: (**a**) CAD model designed in SolidWorks 2016; (**b**) top view with dimensions. Source: Own work.

Tensile tests were proposed to determine the effect of the degradation process on the mechanical properties of the tested material. However, due to the potential use of the analyzed structures as scaffolds for bone defect reconstruction, it should be assumed that, from the perspective of material design and its intended functionality, mechanical properties under compressive loading will be of particular importance. This is due to the nature of the loads occurring in bone tissue, where the implanted structure may be subjected primarily to compressive and cyclic loading. For the uniaxial tensile test, specimens were fabricated with the geometry shown in Figure 1, in compliance with the PN-EN ISO 527:2020 standard [41]. The component was designed as a solid body with a uniform thickness along its entire length, and its dimensions were adapted to normative guidelines. Due to technological limitations and research assumptions, the specimens were manufactured at a 1:4 scale. The length of the gauge section was 15 mm, the width was 2.5 mm, and the thickness was 1 mm.

**Figure 2:**
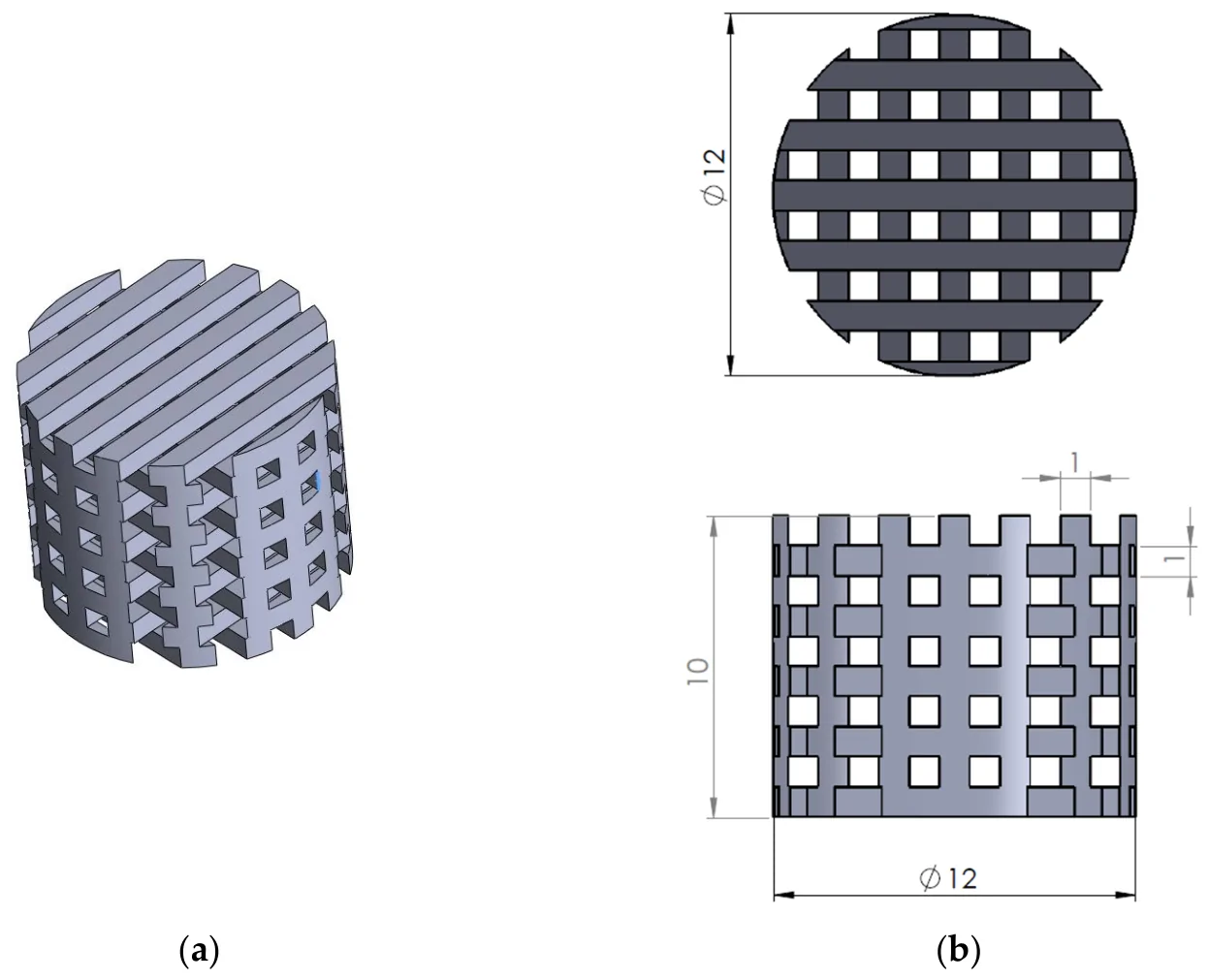
PLLA scaffold 3D model: (**a**) model designed in SolidWorks CAD software; (**b**) side and top views with dimensions. Source: Own work.

For this reason, a detailed analysis of the mechanical test results for the tested material was presented for the proposed mesh structure (Figure 2). The selected geometry is characterized by a significant contact area and a developed surface area of the structure, which may be significant for its functioning in the tissue environment. This developed surface area can influence both the way loads are transferred and distributed, as well as the processes occurring at the material–tissue interface, including the possibility of the structure’s integration with the surrounding tissue. Analysis of the mechanical behavior of this geometry, especially under compression conditions, allows for a better assessment of its suitability for potential use as a scaffold for filling bone defects and to determine the extent to which the degradation process affects the preservation of its mechanical properties.

For the compressive strength testing, a cylindrical scaffold was designed, the 3D model of which is presented in Figure 2. The height of the structure was 10 mm, and its diameter was 12 mm. The spatial geometric model comprised an arrangement of parallel layers with a uniform thickness of 1 mm, oriented in two mutually perpendicular directions. This configuration allowed for the attainment of a regular pore network with dimensions of 1 mm × 1 mm.

The adopted 3D-printing technological parameters were in accordance with the guidelines for the PLLA material: nozzle temperature of 210 °C, build plate temperature of 50 °C, printing speed of 50 mm/s, travel speed (idle nozzle movement) of 30 mm/s, and an infill density of 100%. The manufacturing accuracy was ±0.2 mm.

### 2.3. In Vitro Degradation Studies

Poly(L-lactic acid) degradation studies were conducted in a PBS solution (pH 7.4), which serves as a standard medium for simulating physiological conditions and ensures stable hydrolysis conditions for polyesters. Sample mass changes were monitored at intervals of 0, 1, 7, 15, 30, 45, 60, 75, 90, 120, 150, and 180 days, selected to reflect three characteristic stages of PLLA degradation: early hydration (0–15 days), a period of structural reorganization associated with secondary crystallization (30–90 days), and an advanced degradation phase with more stable kinetics (120–180 days). During the initial weeks, frequent measurements enabled the analysis of the rapid water absorption phase accompanied by minimal mass changes, whereas in the intermediate period, they allowed for the precise recording of transient mechanical property stabilization and the critical moment of stiffness loss. In the final phase, longer intervals were applied due to the slower rate of hydrolysis.

For each time point, *n* = 4 replicates were performed for mass and tensile measurements, and *n* = 3 for compression, which ensured adequate data representativeness along with high reproducibility of the specimens fabricated via FDM. Low standard deviations confirmed the homogeneity of the material and the adequacy of the adopted sample size, while the observed changes—particularly in the 60–90 day interval—reflected actual degradation processes rather than measurement fluctuations.

To ensure conditions closely mimicking the physiological environment of the body, degradation tests were conducted in an Avantgarde Line BD115 incubator (BINDER GmbH, Tuttlingen, Germany) maintaining a constant temperature of 37 °C. The specimens were stored individually in sterile, airtight polypropylene (PP) containers filled with 15 mL of PBS solution. The mass loss of the material ($∆m\left[\%\right]$) was determined based on Equation (1):

(1) $$∆m\left[\%\right]=\frac{m_{0}-m_{t}}{m_{0}}*100\%$$

where $m_{0}$—is the initial mass of the sample, and $m_{t}$—is the mass of the dry sample after a specified degradation time t.

The water content in a sample was measured by the gravimetric method. The sample (wet weight $m_{w}$) was dried in a vacuum oven (105 °C, 24 h) to a low weight (dry weight $m_{t}$). The measurement was repeated four times (*n* = 4) for each time group; measurement uncertainty is ±0.3%.

During the experiment, the pH of the incubation medium was monitored periodically prior to each medium replacement using an Elmetron CPI-505 pH meter (Elmetron, Zabrze, Poland). Furthermore, the PBS solution was regularly replaced every two weeks to maintain stable environmental conditions.

### 2.4. Mechanical Property Testing

The mechanical property testing of the poly(L-lactic acid) included uniaxial tensile and compression tests, which were conducted using a ZwickRoell EPZ 005 universal testing machine (ZwickRoell GmbH & Co. KG, Ulm, Germany). The tensile tests were performed at a constant crosshead speed of 15 mm/min, whereas the compression trials were carried out at a speed of 5 mm/min. In both cases, measurements were taken after 0, 1, 7, 15, 30, 45, 60, 75, 90, 120, 150, and 180 days of sample incubation in the phosphate-buffered saline (PBS) solution.

In the tensile test, four specimens were evaluated for each time point. The recorded force–displacement characteristics were utilized to determine Young’s modulus (E), tensile strength ($R_{m}$), and yield strength ($R_{p0.2}$). Young’s modulus was calculated based on the slope of the initial, linear section of the stress–strain curve within the elastic deformation range. Compression tests were carried out on porous scaffolds, analyzing three specimens for each degradation time point. Based on the obtained plots, Young’s modulus (E), compressive strength ($R_{e}$), and maximum force ($F_{max}$) were determined. The results are presented as arithmetic mean values.

### 2.5. Fourier-Transform Infrared Spectroscopy (FTIR)

The chemical structure of poly(L-lactide) was analyzed using Fourier-transform infrared spectroscopy in attenuated total reflection (FTIR-ATR) mode with a Nicolet iS50 spectrometer (Thermo Fisher Scientific, Waltham, MA, USA). The samples analyzed were those previously used in a static tensile test, which allowed for an assessment of the effects of mechanical loading and the degradation process on structural changes in the material. Spectra were recorded in the wavenumber range from 4000 to 400 cm^−1^, at an optical resolution of 4 cm^−1^, averaging 16 scans for each measurement. The number of repetitions for each time point was *n* = 4. The obtained spectra were analyzed to identify characteristic absorption bands attributed to the vibrations of PLLA functional groups and to compare the changes occurring in the material during incubation in a PBS environment.

### 2.6. Scanning Electron Microscopy (SEM) Analysis

Surface morphology evaluation of the PLLA scaffolds was performed using field emission scanning electron microscopy (SEM, JEOL JSM-7600F, JEOL Ltd., Tokyo, Japan). The SEM investigations enabled the assessment of changes in the scaffold structure and its homogeneity resulting from the degradation process occurring in the PBS solution. Based on the obtained mechanical characterization results, specimens from four time points were selected for microscopic examinations: before degradation (0 days) and after 90, 120, and 180 days of incubation in PBS. Prior to observation, the samples were coated with a 6 nm thick conductive chromium layer using a vacuum sputtering system (Quorum Q150T S, Quorum Technologies, Laughton, UK). The surface of the samples was analyzed at a magnification of 250× with an accelerating voltage of 2 kV. The number of repetitions for each time point was *n* = 3.

## 3. Results

### 3.1. Mass Changes Analysis

The mass loss of PLLA scaffolds during incubation in PBS primarily results from the hydrolytic degradation of ester bonds (bulk hydrolysis), which leads to scission and shortening of polymer chains. In the initial stage, this process causes a decrease in molecular weight, whereas a measurable mass loss occurs only when short, water-soluble oligomers capable of diffusing into the solution are formed [42]. In the case of porous scaffolds, physical phenomena also play a significant role, including water absorption, the leaching of dissolved compounds, and morphological changes (e.g., structural microcracks).

**Figure 3:**
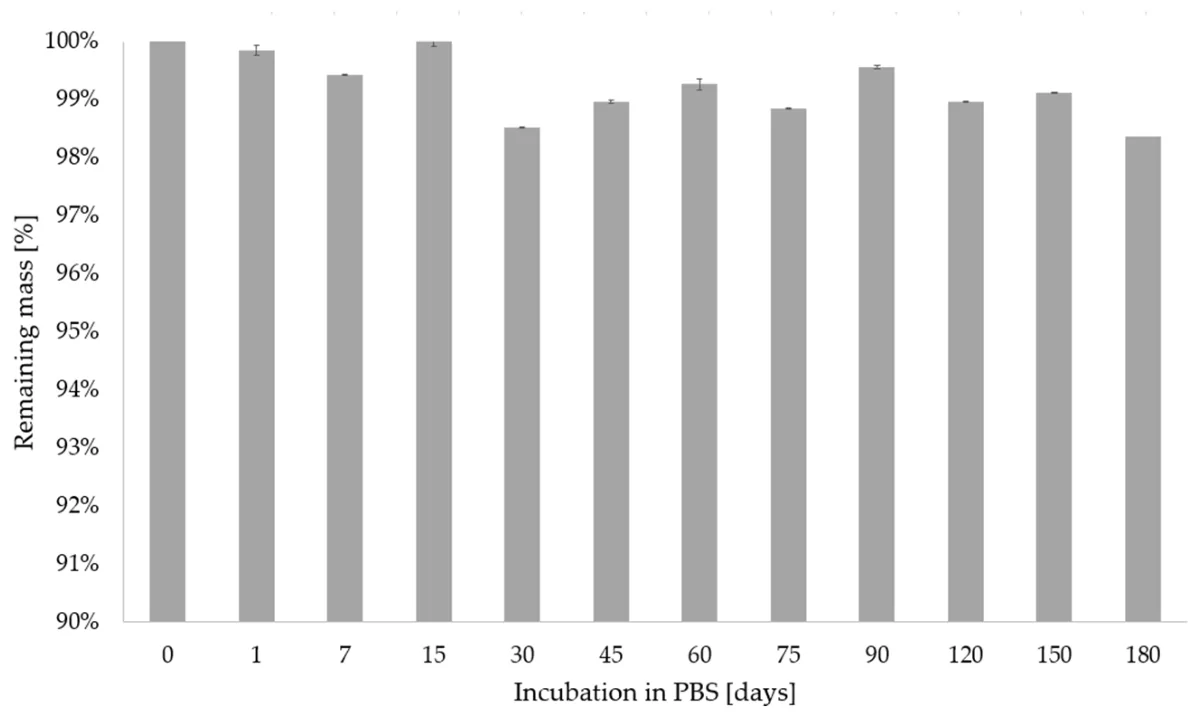
Results of the relative mass loss analysis during PLLA degradation.

Figure 3 shows the relative mass loss compared to the previous measurement. These data allow us to determine the intensity of mass loss at different stages of degradation. In addition to the analysis of total mass loss, percentage changes in mass loss were observed in subsequent measurement periods. During the initial days of incubation (1–15 days), the remaining mass of the specimens was stable and ranged between 99.42% and 100%, which is typical for the early stage of material hydration when water penetrates the porous architecture of the scaffold (Figure 3). This may be attributed to local variations in the degree of hydration and the efficiency of specimen drying prior to weighing.

A distinct decrease in the remaining mass (down to 98.52%) was observed between days 15 and 30 of incubation in PBS. This may indicate a transition from the first stage of degradation, where chain scission occurs predominantly without mass loss, to the second stage, in which short, soluble macromolecular fragments are generated and diffuse into the incubation medium. In the 30–75 day period, the sample mass values exhibited minor fluctuations within the range of 98.85% to 99.27%. This stabilization may result from the overlap of progressive hydrolysis and secondary structural rearrangements of PLLA, such as chain reorganization and a local increase in the degree of crystallinity. An increased crystalline phase content can temporarily restrict water diffusion into the material interior and slow down the erosion process. Furthermore, in porous structures, differences in drying efficiency and the presence of water entrapped within micro- and macropores can generate slight fluctuations in the results, despite the globally progressive degradation trend. In the 75–180-day interval, a further, gradual progression of PLLA matrix degradation was observed. The remaining mass values ranged from 98.36% to 99.56%, showing slight variations determined by the simultaneous occurrence of hydrolysis and polymer structural changes. Nonetheless, the overall trend of the curve clearly indicates the successive degradation of poly(L-lactic acid), as confirmed by the lowest remaining mass (98.36%) recorded after 180 days of incubation in PBS.

During the experiment, the pH of the incubation medium was routinely monitored prior to each replacement and remained stable at 7.4 (±0.2) throughout the 180-day period. Furthermore, the PBS solution was regularly replaced every two weeks to maintain stable environmental conditions and prevent local acidification from degradation byproducts.

### 3.2. Mechanical Properties of PLLA Scaffolds Before and After Degradation

**Figure 4:**
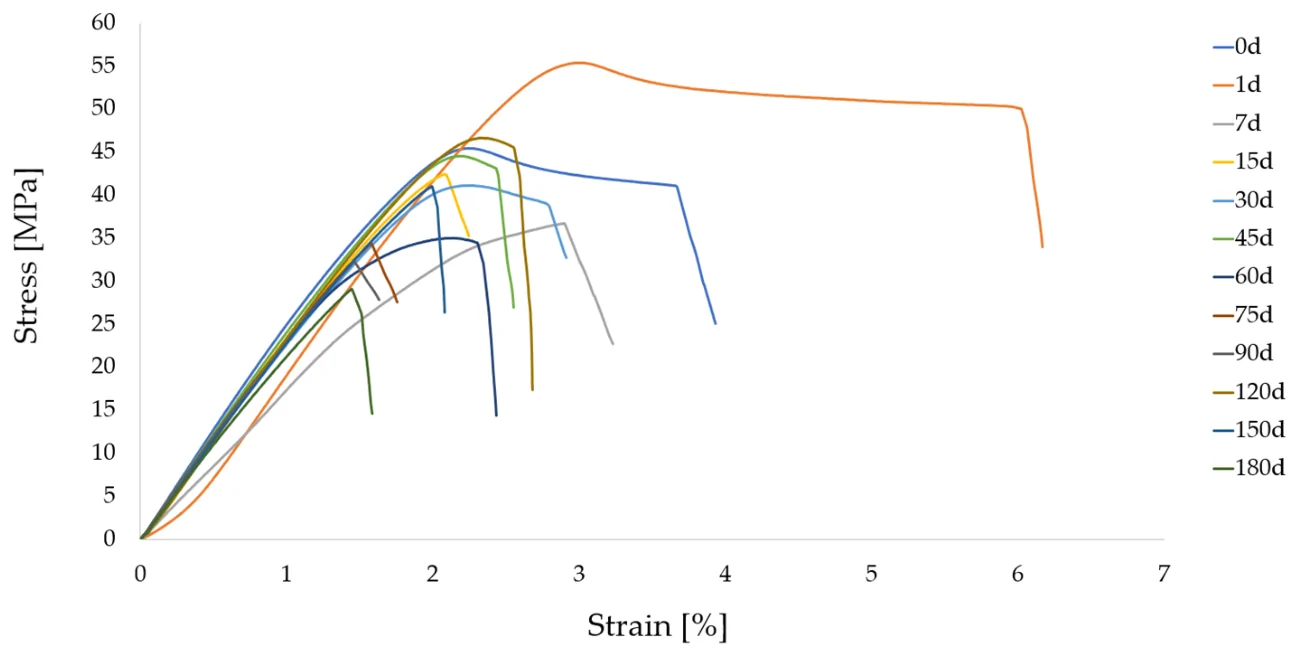
Stress–strain characteristics obtained from the uniaxial tensile test.

The mechanical properties of poly(L-lactic acid) in its initial state and at individual stages of degradation were characterized based on the results of uniaxial tensile and compression tests. The profile of the obtained stress–strain curves serves as the basis for evaluating the impact of the degradation process on the material behavior under load, as well as for determining parameters describing its strength and deformation properties. Figure 4 illustrates a characteristic stress–strain relationship. Based on this curve, the values of Young’s modulus (E), tensile strength ($R_{m}$), and the offset yield strength determined at 0.2% plastic strain ($R_{p0.2}$) were defined.

The stress–strain curve in the initial loading range exhibited similar behavior within the elastic strain range; however, the subsequent mechanical response revealed a complex, non-monotonic trend during the incubation period (Figure 4). The obtained mechanical parameters indicate a noticeable impact of the degradation environment on the mechanical properties of PLLA (Table 1).

**Figure 5:**
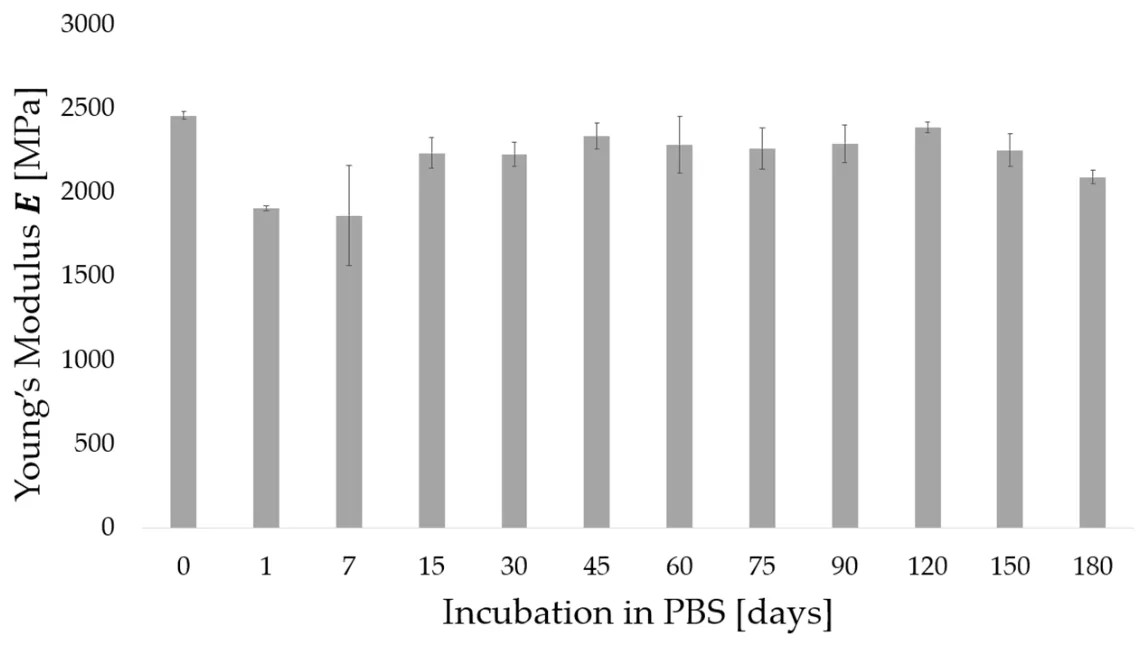
Comparison of Young’s modulus (E) values determined during the uniaxial tensile test of PLLA specimens.

The obtained mechanical parameters indicate a noticeable impact of the degradation environment on the mechanical properties of PLLA (Table 1). Between day 0 and day 1 of incubation, Young’s modulus exhibited a sharp drop from 2458.07 ± 24.29 MPa to 1905.37 ± 16.52 MPa, which is likely associated with the rapid penetration of water molecules into the polymer matrix and the resulting initial plasticization effect (Figure 5). Interestingly, by day 15, the modulus recovered to 2234.59 ± 92.24 MPa. This initial fluctuation can be attributed to the complex interplay between rapid hydration, macromolecular relaxation, and potential solvent-induced rearrangements within the polymer structure during the early stages of incubation. A corresponding shift was observed for the yield strength ($R_{p0.2}$), which dropped significantly from 4.87 ± 0.11 MPa at day 0 to its lowest value of 1.04 ± 0.06 MPa on day 1, before recovering to 3.33 ± 1.67 MPa by day 7. This substantial, temporary drop is consistent with the initial plasticization of the PLLA matrix, where the rapid binding of water molecules temporarily lowers the stress threshold required to initiate irreversible macromolecular sliding. As secondary crystallization progresses and crystalline domains rearrange in the subsequent days, the polymer network is systematically reinforced, restoring the yield strength to its stable baseline range of approximately 4–5 MPa. Following this initial phase, Young’s modulus stabilized, showing minor variations between day 15 and day 150 (remaining within the 2228–2389 MPa range). By day 180, Young’s modulus decreased to its lowest value of 2091.48 ± 42.1 MPa, which was accompanied by a clear reduction in tensile strength ($R_{m}$) to 31.11 ± 2.97 MPa. The recorded long-term tendencies show high agreement with the literature data reported by Polak-Kraśna et al. [43] and Malone et al. [44]. These authors also demonstrated that long-term incubation of PLLA in a PBS environment leads to a successive reduction in the modulus of elasticity and tensile strength, with a simultaneous shortening of the failure strain range, which is a direct consequence of hydrolytic polymer chain scission and the increasing brittleness of the matrix.

**Figure 6:**
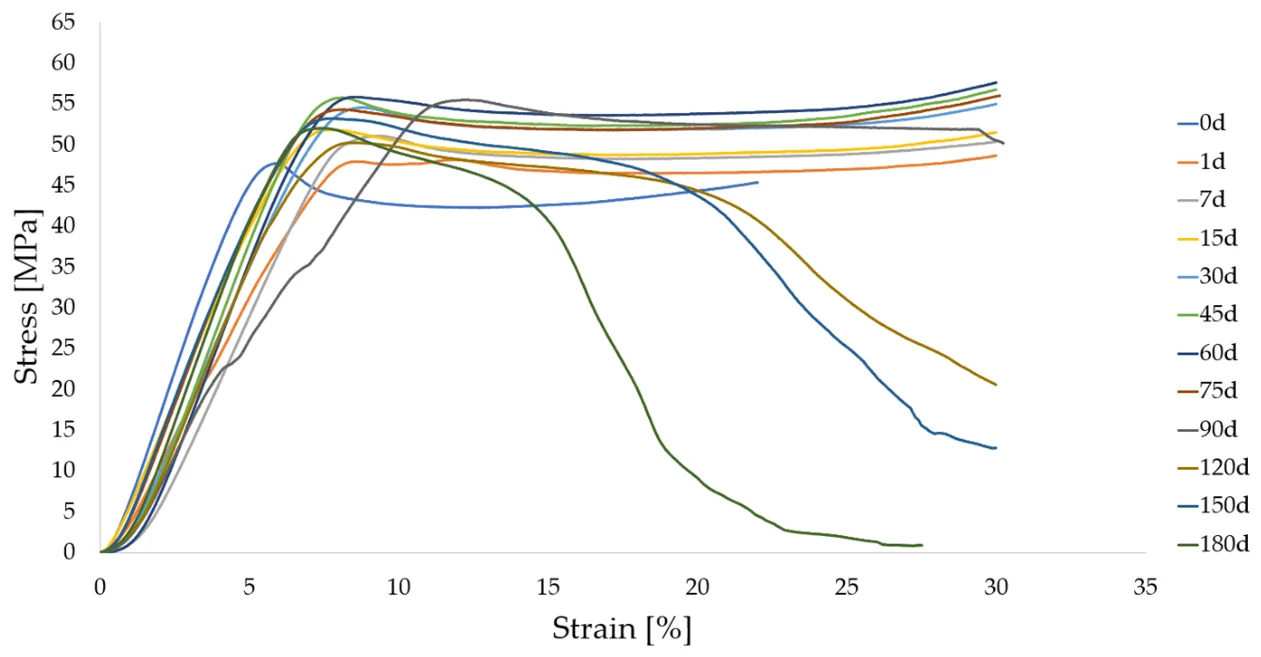
Stress–strain characteristics obtained from the uniaxial compression test.

The results of the uniaxial compression test indicate a varied impact of incubation time on the mechanical properties of poly(L-lactic acid), as confirmed by the data summarized in Table 2 and the stress–strain curves (Figure 6). Up to approximately day 60 of incubation in the PBS medium, the values of the modulus of elasticity and compressive strength did not exhibit a clear downward trend. This suggests a relatively stable behavior of the porous scaffold structure during the initial stage of the degradation process.

**Figure 7:**
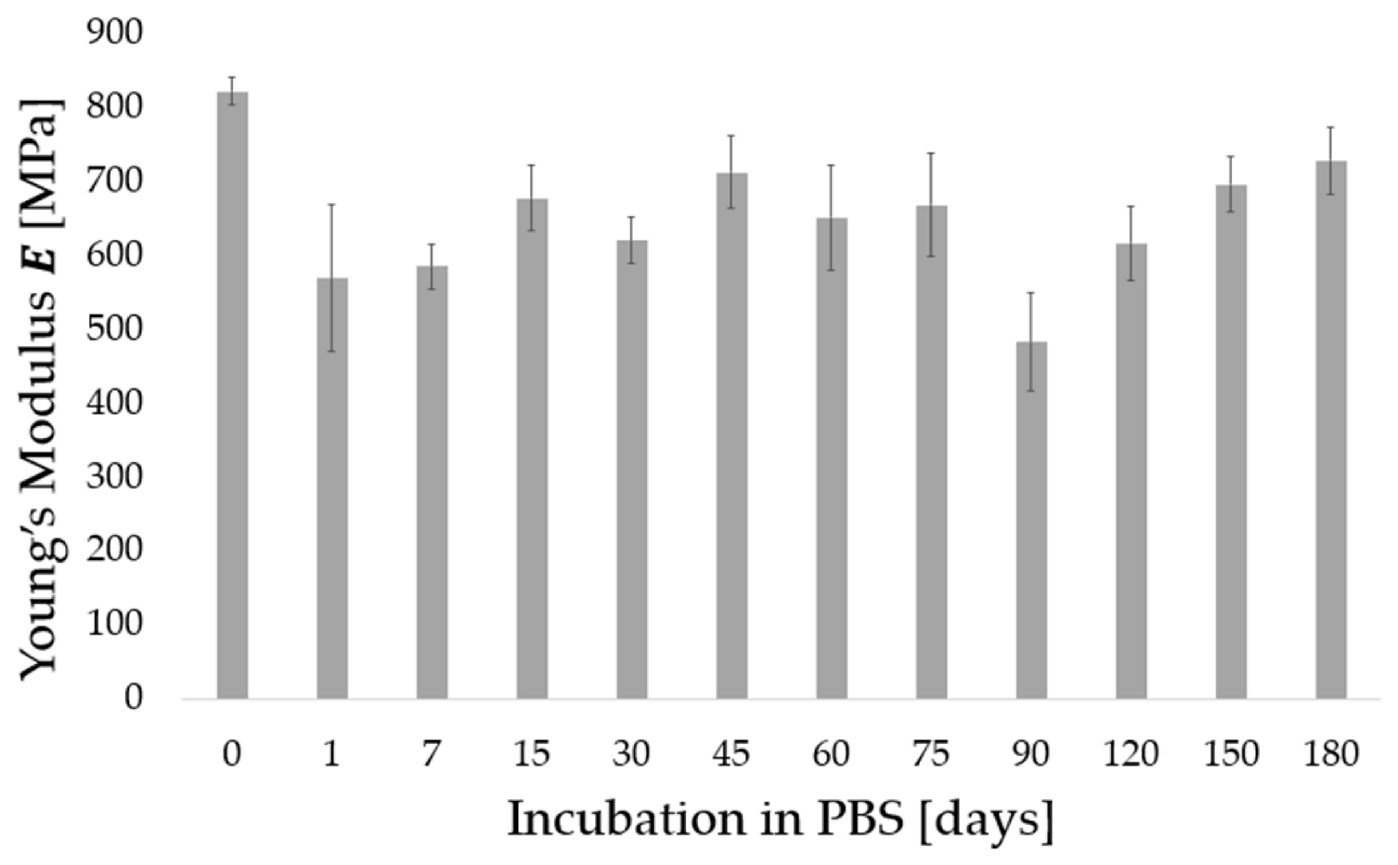
Comparison of Young’s modulus values obtained from the uniaxial compression test of PLLA scaffolds at different degradation stages.

A distinct change in the character of the material’s mechanical response becomes apparent from day 90 of degradation. Interestingly, the compressive Young’s modulus exhibited an apparent increase in the final stages of degradation, rising from 482.06 ± 65.94 MPa on day 90 to 727.07 ± 45.05 MPa on day 180 (Figure 7). This trend correlates directly with the transition of the PLLA matrix from a ductile to a highly brittle state, as evidenced by the altered failure mode of the specimens during compression documented in the post-test photographs included in Table 2. Due to the preferential hydrolytic erosion of the compliant amorphous regions over long-term incubation, the remaining material becomes increasingly dominated by rigid crystalline domains. This structural shift elevates the initial stiffness of the scaffolds, resulting in a higher apparent Young’s modulus. However, this crystal-dominated matrix lacks the capacity for plastic deformation; while the day 90 specimens deform cohesively under load, the highly brittle day 180 scaffolds lose their capacity for uniform deformation and undergo fragmentation during the compressive loading, shattering into the small debris visible in the post-deformation images. In the work of Kuchumov et al. [45], similar studies regarding the evaluation of mechanical property changes in PLLA stents were described. The noted differences in the results are determined solely by the arrangement of PLLA fibers (printing paths) in the fabricated 3D models.

The analysis of the results from both the uniaxial tensile and compression tests clearly confirms the significant impact of the degradation process on the mechanical response of poly(L-lactic acid). This effect is manifested by a successive reduction in strength parameters and a distinct limitation of the material’s capacity for plastic deformation.

### 3.3. Fourier-Transform Infrared Spectroscopy (FTIR) Analysis

**Figure 8:**
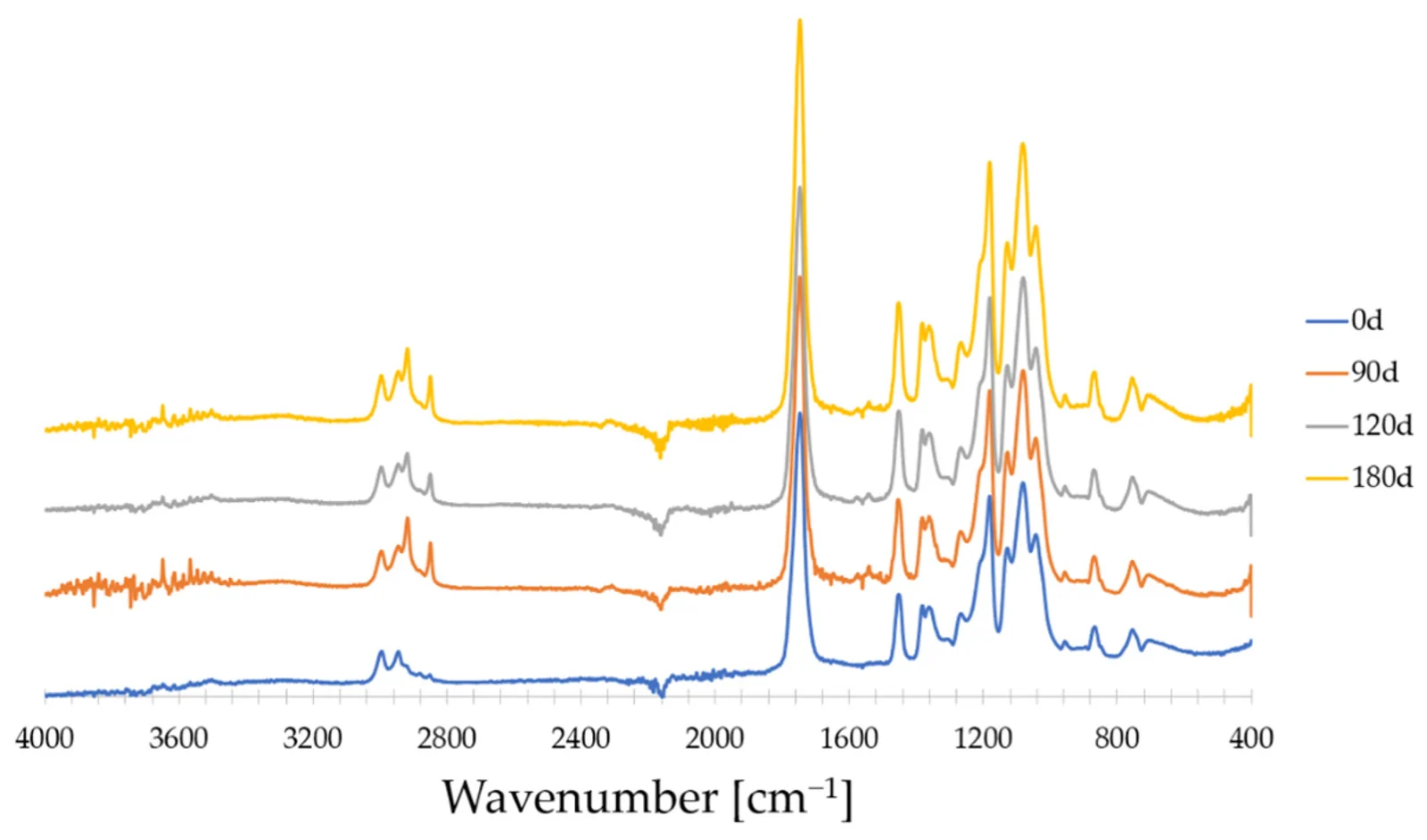
FTIR spectra of poly(L-lactic acid)-based scaffolds at different stages of degradation.

The FTIR spectra of the poly(L-lactic acid)-based scaffolds, recorded in the wavenumber range from 4000 to 400 cm^−1^ after 0, 90, 120, and 180 days of incubation in the PBS, are presented in Figure 8. The spectra exhibit absorption bands characteristic of the individual functional groups of PLLA.

The first distinct absorption bands are the peaks located around 1100 and 1200 cm^−1^, which correspond to the stretching vibrations of C−O bonds characteristic of ester groups in the PLLA structure. Another band, observed at approximately 1380 cm^−1^, is assigned to the bending vibrations of methyl groups ($-CH_{3}$). The intense, sharp peak around 1750 cm^−1^ corresponds to the stretching vibrations of carbonyl groups (C=O). In the range from 2850 to 2950 cm^−1^, bands associated with the stretching vibrations of C−H bonds originating from methyl ($-CH_{3}$) and methine (−CH) groups are visible. Additionally, a broad band corresponding to the stretching vibrations of hydroxyl groups (−OH) is observed around 3000 cm^−1^. The obtained FTIR analysis results show high consistency with literature data, in which analogous bands of ester, methyl, carbonyl, and hydroxyl groups are identified for the PLLA matrix. A similar position and intensity of the characteristic peaks were confirmed in the work of Rahaman et al. [46], proving the correctness of functional group identification in the studied material. Furthermore, according to literature reports, the dominant degradation mechanism of poly(L-lactic acid) is the random cleavage of ester bonds via hydrolysis. This process leads to the formation of lactic acid (and its oligomers), which is naturally metabolized by the body and exhibits no toxic effects, confirming the biocompatibility of PLLA and its suitability for tissue engineering [17].

### 3.4. Scanning Electron Microscopy (SEM) Surface Morphology Evaluation

**Figure 9:**
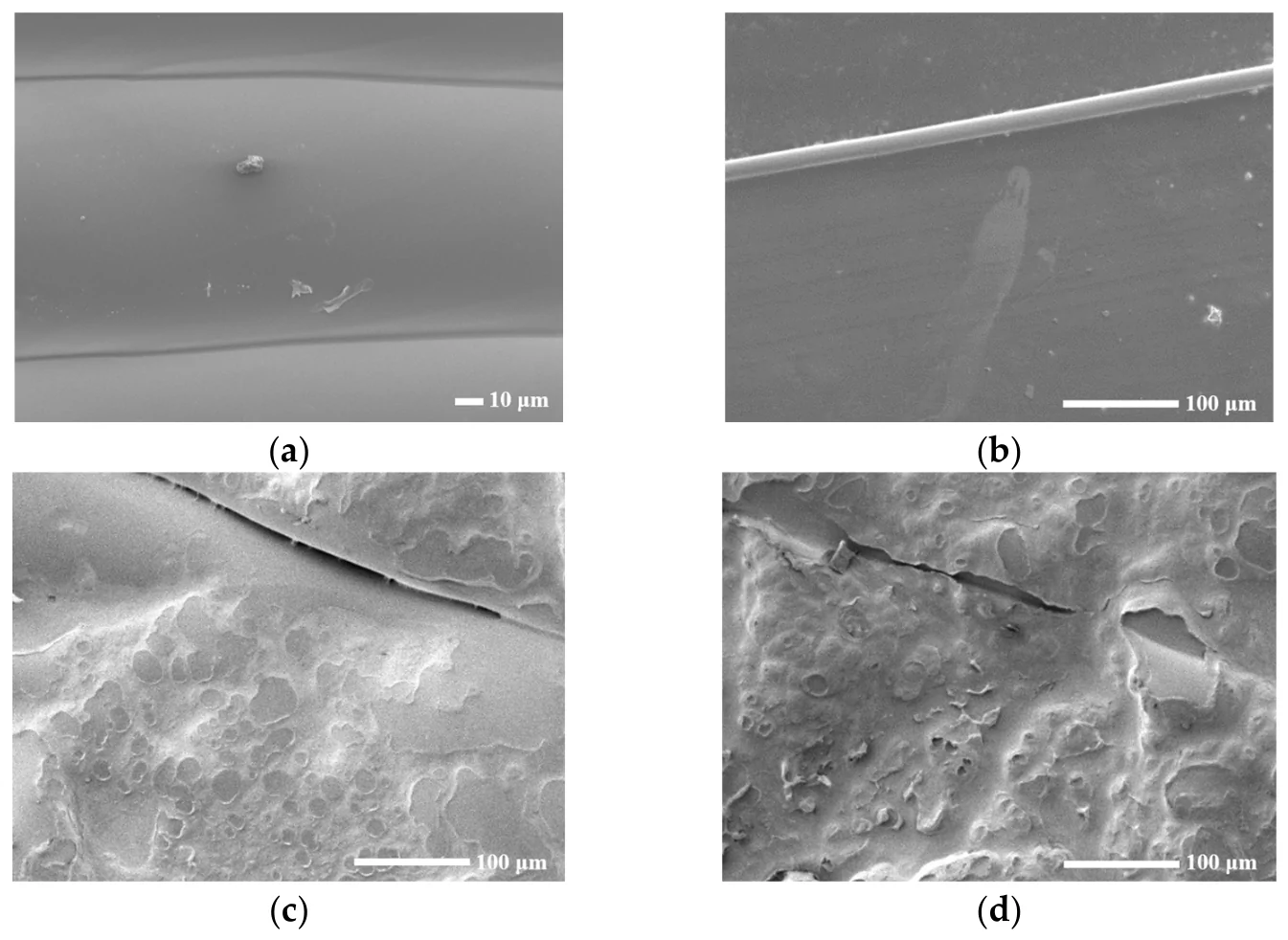
SEM micrographs of PLLA scaffold surfaces: (**a**) before degradation; (**b**) after 90 days; (**c**) after 120 days; and (**d**) after 180 days of incubation in PBS.

The evaluation of the structure of polymeric materials after the incubation period is crucial from the perspective of their potential medical applications. Figure 9 presents SEM micrographs of the PLLA scaffold surfaces recorded for four experimental time points.

Before degradation (Figure 9a), the scaffolds were characterized by a smooth, homogeneous surface. A few surface artifacts resulted directly from the specifics of the 3D-printing process in FDM technology. The initial structure of the material was compact and stable. After 90 days of incubation in the PBS solution (Figure 9b), no significant macroscopic changes were observed either. Nevertheless, the first irregularities began to appear in the surface layer. This phenomenon signals the initial phase of ester bond hydrolysis, which at this stage does not yet disturb the structural integrity of the scaffold. A clear increase in surface roughness, depressions, and micropores was revealed in the material after 120 days of degradation (Figure 9c). This indicates the progress of hydrolytic degradation, which leads to a reduction in the molecular weight of the polymer. After 180 days of incubation (Figure 9d), the material structure underwent permanent, advanced destruction. Numerous pits, irregular losses of the polymer matrix, a highly developed surface topography, and structural cracks were observed. After both 120 and 180 days of incubation in the PBS solution, the surface morphology indicates a deep destabilization of the polymer matrix. The obtained SEM micrographs fully correlate with the results of the mechanical tests, in which a rapid decrease in the strength parameters of the PLLA scaffolds was recorded after the same incubation periods.

### 3.5. Coupled Analysis of Mechanical and Physicochemical Property Evolution of PLLA

The proposed material model was solved by seeking relationships between the mechanical properties of the material and the degradation time.

**Figure 10:**
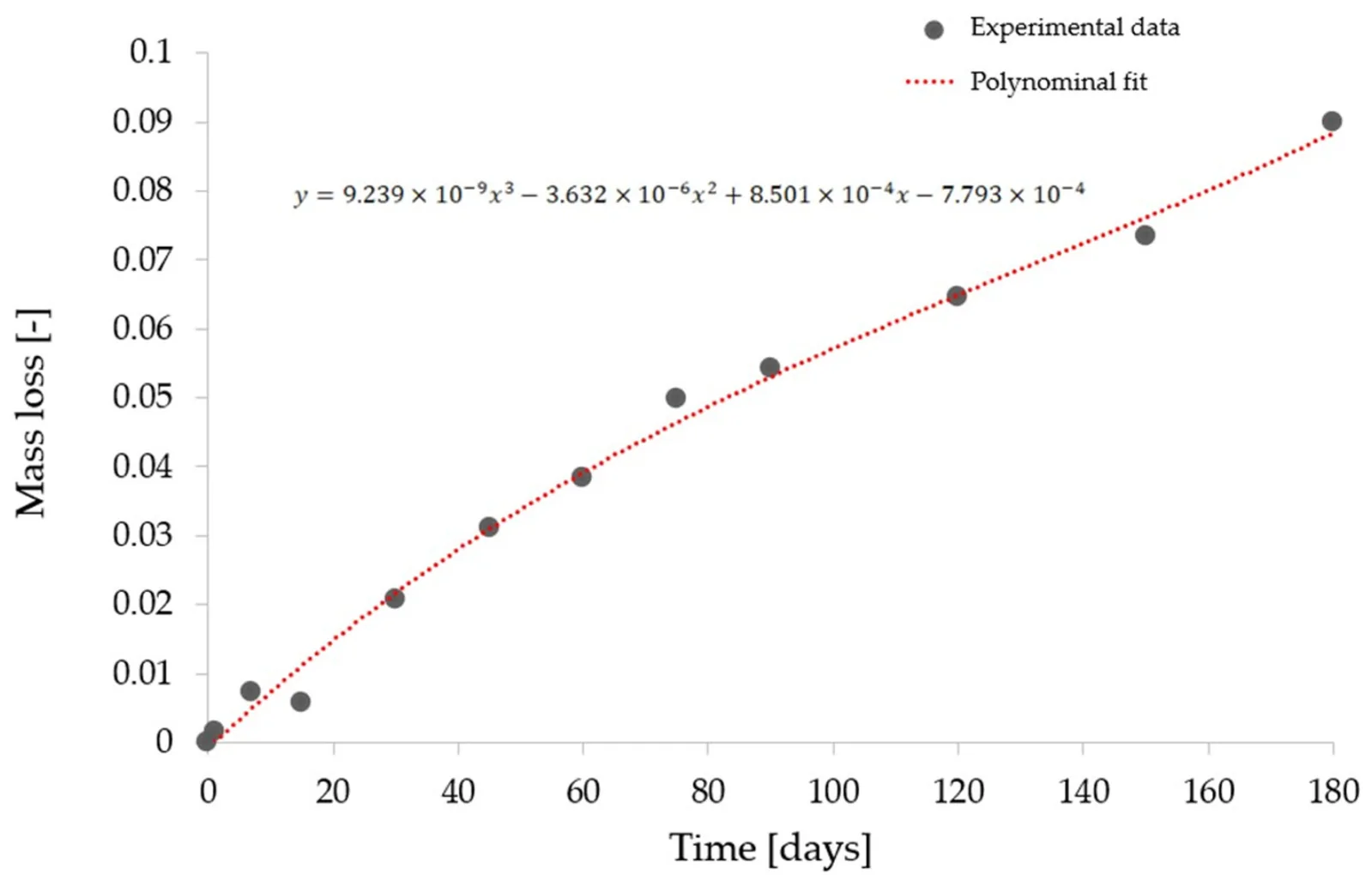
Mass loss over time.

The mass loss (Figure 10) also exhibits a general upward trend, reaching approximately 8.98% after 180 days. Despite minor non-monotonicities, which can be attributed to measurement errors or local structural variations, this profile confirms the dominance of hydrolytic bulk degradation without rapid surface erosion.

**Figure 11:**
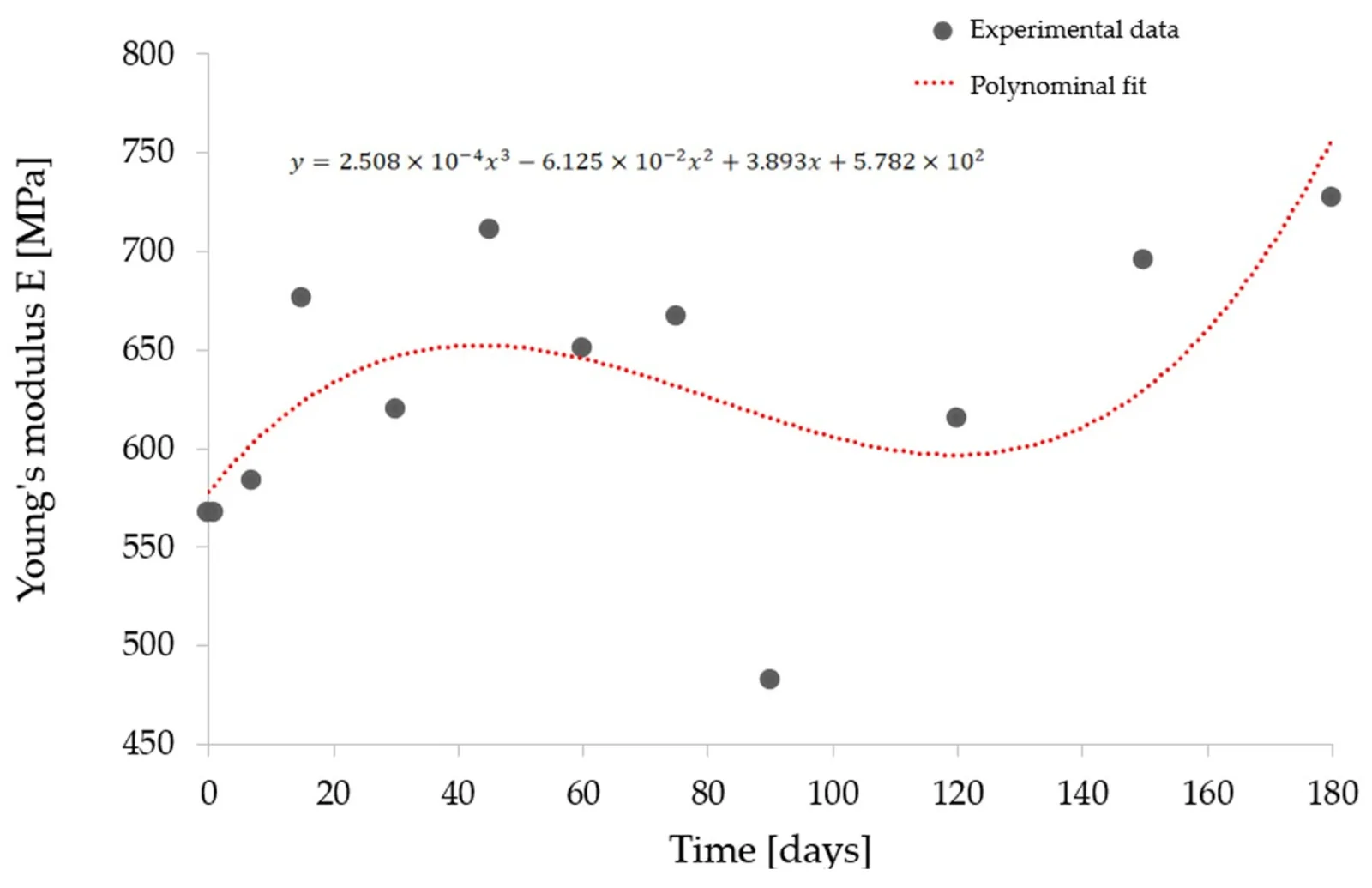
Evolution of Young’s modulus.

The most complex behavior is observed in the case of mechanical properties in compressive tests (Figure 11). Young’s modulus does not change monotonically—it initially increases, then undergoes fluctuations, and subsequently increases again. This indicates the coexistence of two competing mechanisms: secondary crystallization, which increases the stiffness of the material, and the degradation of polymer chains, which leads to its weakening.

**Figure 12:**
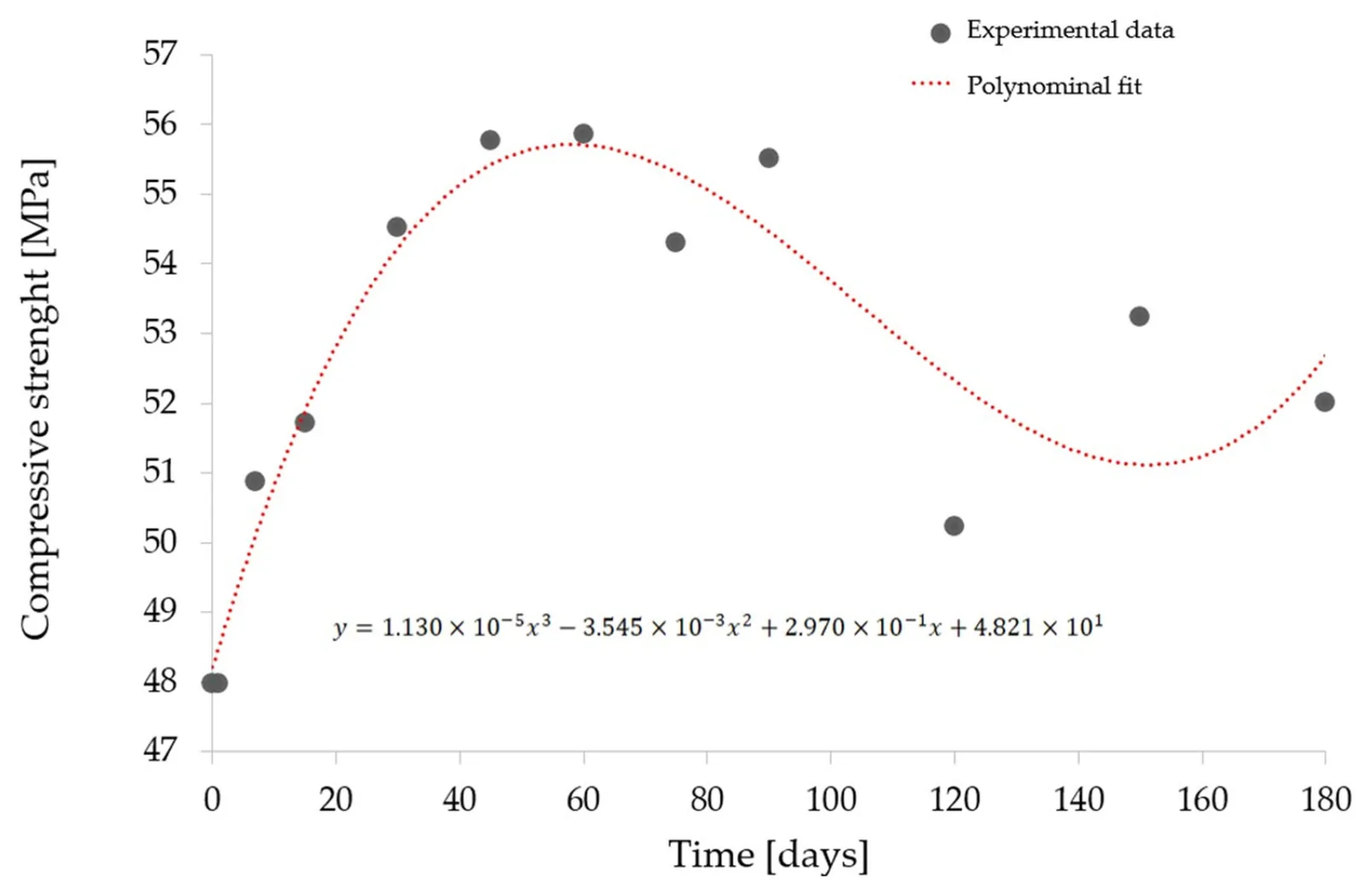
Evolution of compressive strength.

The compressive strength remains relatively stable, meaning that the material retains its load-bearing capacity for a long time. Around day 60, significant strengthening occurs, after which the further process of structural rearrangement leads to a significant weakening of the structure (Figure 12).

**Figure 13:**
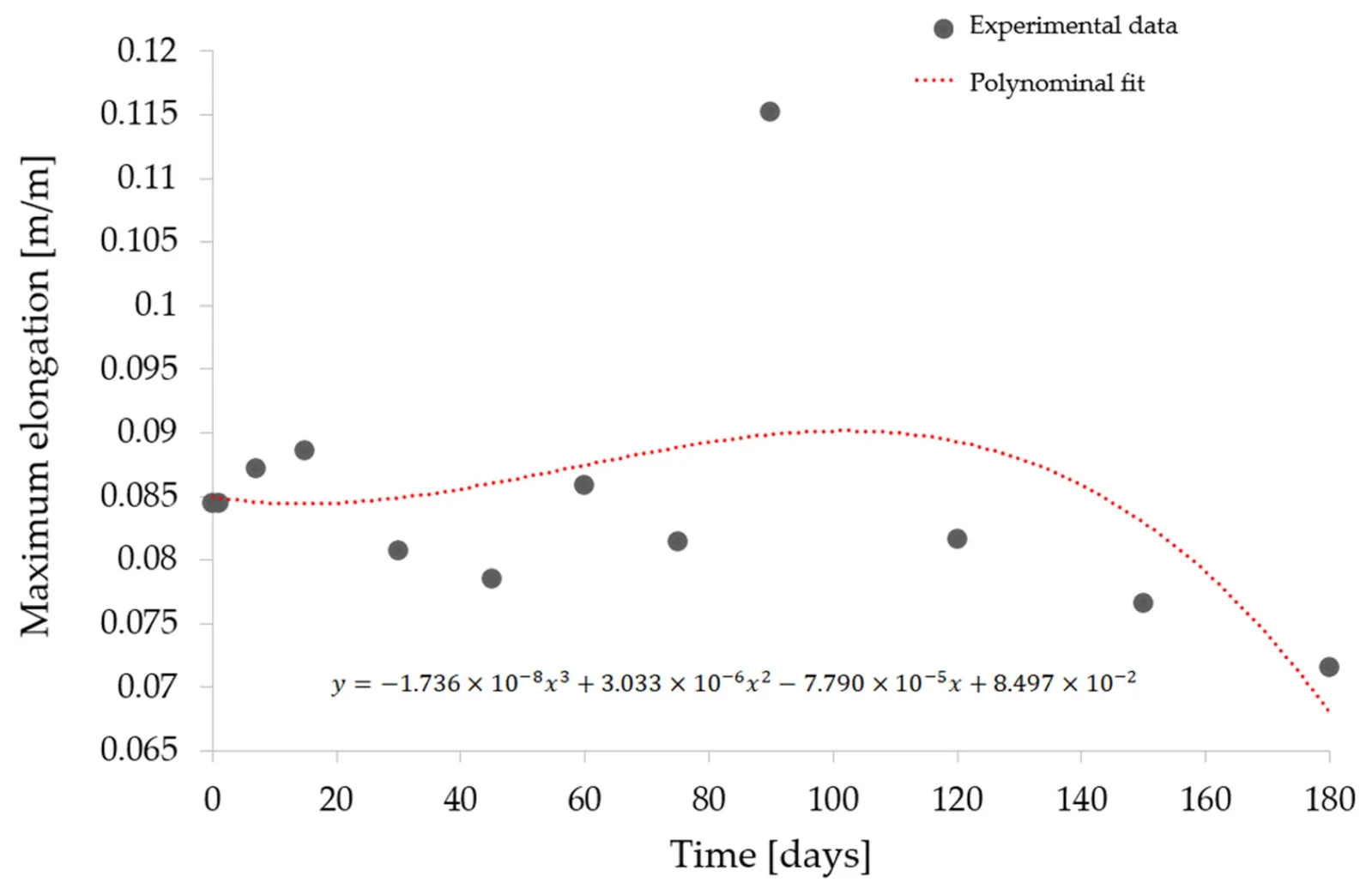
Evolution of maximum elongation.

In turn, the maximum strain shows an initial increase followed by a decrease, indicating a transition in the material from a more ductile to a more brittle state (Figure 13).

Overall, the results confirm that the mechanical properties of the material are directly linked to the degradation progress and water absorption.

## 4. Discussion

The mechanical response of the PLLA specimens incubated in PBS exhibited significant changes over the studied degradation periods (0–180 days). The tensile strength initially increased, reaching a maximum around day 60, followed by a distinct decrease. In comparison, Young’s modulus reached a minimum around day 90, after which a partial recovery was observed.

Such behavior indicates that the mechanical response is controlled by competing degradation and structural reorganization processes rather than by simple monotonic chain scission.

The Integrated Difference Spectrum (IDS) method provides a quantitative approach to analyzing FTIR spectral changes occurring between two material states, based on integrating the absolute value of the absorbance difference as a function of the wavenumber. Formally, it is defined as the sum of integrals over selected spectral ranges.

The extension of the IDS by partitioning into spectral ranges was performed based on Equation (2):

(2) $$IDS_{i}=\int_{\nu_{1}^{i}}^{\nu_{2}^{i}}|A_{t_{2}}-A_{t_{1}}|d\nu$$

where $A_{t_{1}}\;(\nu )$ and $A_{t_{2}}\;(\nu )$ denote the FTIR spectra at two different time points, respectively, while the index i refers to the separated spectral regions corresponding to specific functional groups.

This approach enables a transition from a pointwise analysis based on individual absorption bands to a global evaluation of structural changes within the entire spectrum or its functionally relevant fragments.

From a physicochemical perspective, the IDS is interpreted as a measure of the “spectral distance” between two material states, as it integrates all alterations occurring in the shape, intensity, and position of the absorption bands. In contrast to classical FTIR indices based on the intensity ratios of selected peaks, this method simultaneously accounts for band broadening, asymmetry, maximum shifts, and spectral background alterations. Consequently, the IDS is particularly useful for materials in which chemical and physical processes occur simultaneously, leading to overlapping changes in the FTIR signal.

In the case of biodegradable polymers such as PLLA, hydrolytic degradation leads to simultaneous changes in multiple chemical systems, including the cleavage of ester bonds, the formation of −OH and −COOH terminal groups, alterations in the degree of crystallinity, the reorganization of amorphous phases, and water absorption. Each of these processes affects not only the intensity of selected bands but also their width and shape, which complicates a straightforward interpretation based on classical peak indices. The IDS integrates all these effects, enabling a quantitative evaluation of the total chemical structure alteration of the material as a function of time.

Analysis within selected spectral ranges additionally allows the observed changes to be assigned to specific chemical mechanisms. The 2800–3000 cm^−1^ region corresponds to the stretching vibrations of C−H bonds in the polymer backbone and provides information on conformational changes and chain mobility. The 3200–3500 cm^−1^ range is associated with −OH groups participating in hydrogen bonds and reflects the degree of hydration and the development of interchain interactions. The 3500–3800 cm^−1^ region is assigned to free hydroxyl groups and weakly bound water, serving as a direct indicator of advanced hydrolysis and an increasing number of chain ends. The 1500–1800 cm^−1^ range encompasses the vibrations of carbonyl (C=O) groups, which are strongly linked to the degradation of ester bonds and the reorganization of the crystalline-amorphous structure, whereas the 1500–1600 cm^−1^ band corresponds to carboxylate ($COO^{-}$) groups, which are direct products of hydrolysis.

**Figure 14:**
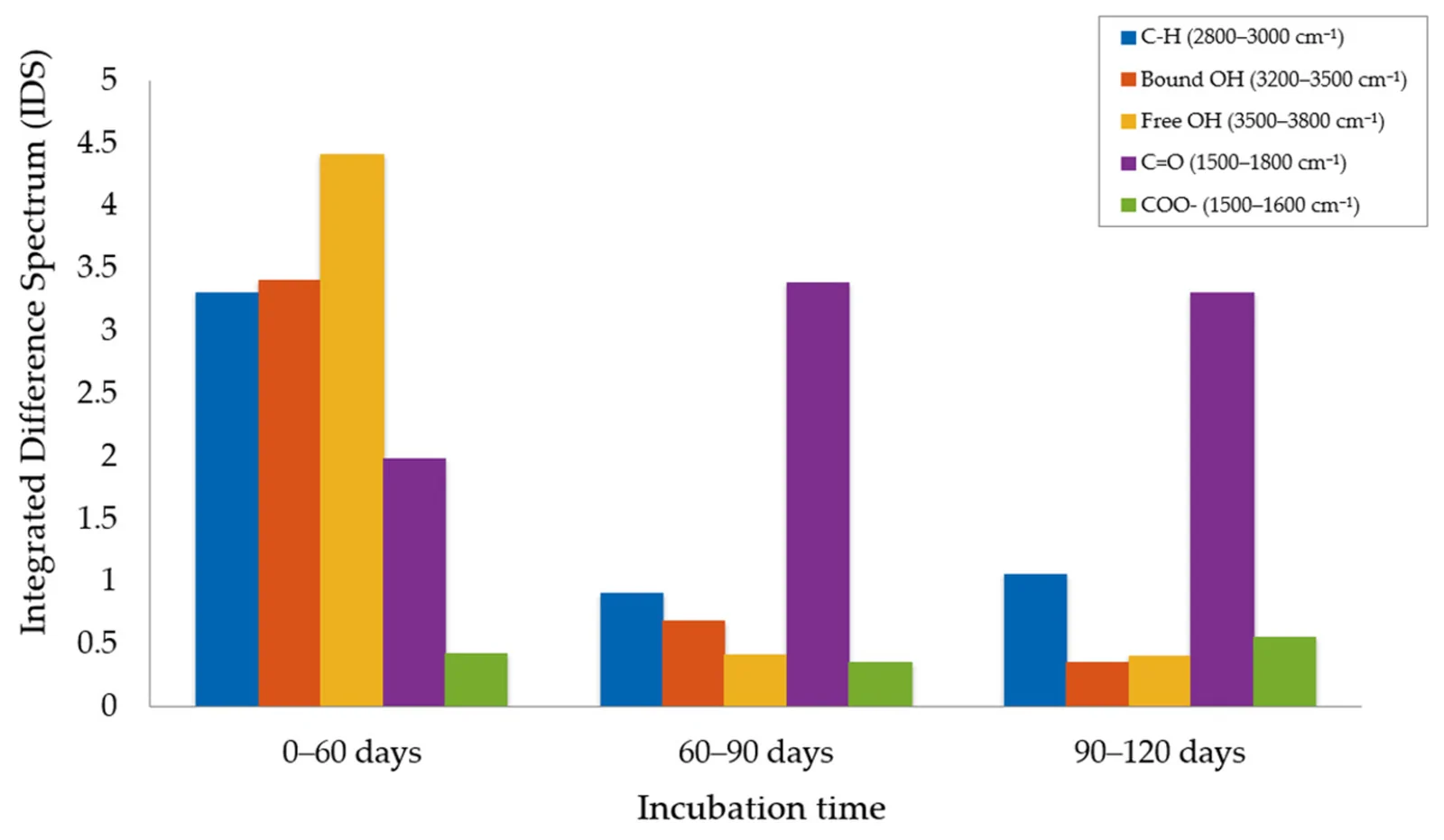
IDS values for different ranges of wavenumbers.

Therefore, the application of the IDS enables the simultaneous capture of both the global intensity of structural changes and their chemical specificity with respect to individual degradation mechanisms (Figure 14). Consequently, this method constitutes a sensitive tool for analyzing the aging and degradation processes of polymeric materials, allowing for the correlation of spectral alterations with physicomechanical properties, such as the decrease in Young’s modulus, changes in strength, or microstructure reorganization.

The analysis of IDS values as a function of time allows not only for the identification of the chemical mechanisms of PLLA degradation but also for directly linking these alterations to changes in the mechanical properties of the material, particularly Young’s modulus, strength, and deformation capacity.

In the first stage (0–60 days), changes within the 1500–1800 cm^−1^ range dominate (IDS = 4.3704), indicating an intensive rearrangement of carbonyl (C=O) groups, which is characteristic of the initial phase of ester bond hydrolysis and the reorganization of the amorphous-crystalline structure. During the same period, relatively high activity is observed in the C−H stretching region (2800–3000 cm^−1^), suggesting conformational changes of the chains, yet without their deep fragmentation. Mechanically, this corresponds to a phase where the material may even exhibit a local increase in strength, resulting from structural reorganization and possible secondary crystallization, while simultaneously maintaining a relatively high integrity of the entanglement network. This is consistent with the short-term stabilization or even the strength maximum frequently observed in PLLA during the initial phase of degradation.

In the second stage (60–90 days), a distinct shift in the dominant chemical mechanism occurs. The largest contribution shifts to the 1500–1600 cm^−1^ range (IDS = 3.3606), corresponding to the formation of carboxylate ($COO^{-}$) groups, which unambiguously indicates advanced hydrolysis and the accumulation of degradation products. Simultaneously, a decrease in the contribution of changes within the carbonyl (C=O, 1500–1800 cm^−1^) and alkyl (C−H, 2800–3000 cm^−1^) regions is observed, suggesting a loss of chain structure continuity and a significant reduction in molar mass. This stage corresponds to a critical moment in the evolution of mechanical properties, where a rapid drop in Young’s modulus and strength takes place. The reduction in chain length and the decrease in the number of entanglements lead to a loss of the material’s capacity to effectively transfer stresses, which manifests as the minimum stiffness observed during this period. Concurrently, the increased presence of polar groups promotes water sorption, which further plasticizes the material and exacerbates the weakening of its mechanical properties.

In the third stage (90–120 days), a further stabilization of degradation processes is observed, with a sustained high IDS value of the carboxylate ($COO^{-}$) band, indicating the fixation of hydrolysis products within the material structure. Simultaneously, the relative contribution of the 3500–3800 cm^−1^ range increases, demonstrating an enhanced content of free hydroxyl groups and an increase in water absorption. In this stage, the material is already characterized by low structural cohesion, and its mechanical properties undergo further degradation, though often with lower dynamics than in the transition phase. Partial reorganization of chain fragments and local crystallization may also occur, leading to a partial stabilization of the modulus of elasticity, while the strength continues to decline.

In conclusion, the observed changes in IDS values unambiguously correlate with the evolution of the mechanical properties of PLLA: the initial structural reorganization can lead to transient stabilization and a strength increase; subsequently, during the 60–90 day phase, critical weakening occurs due to intensive hydrolysis and molar mass loss; finally, the dominant presence of degradation products and a secondary mechanical–structural stabilization are observed in the terminal stage.

## 5. Conclusions

The obtained initial values of Young’s modulus (2.46 GPa) and tensile strength (45.5 MPa) are in good agreement with literature data for semicrystalline PLLA fabricated via the FDM method. For this type of material, typical values of the modulus of elasticity fall within the range of 2–4 GPa, whereas the tensile strength ranges from 40 to 60 MPa [43,47]. The slightly reduced mechanical parameters compared to bulk PLLA result from the presence of porosity and interlayer discontinuities inherent to additive manufacturing technology.

The recorded mass loss after 180 days of incubation in PBS (8.98%) is consistent with other studies on the hydrolytic degradation of high-crystallinity PLLA. In the initial degradation phase, the dominant process is the reduction in molecular weight, while the actual mass loss remains limited due to the hindered water penetration into the ordered crystalline regions [43].

The observed transition of the material into a brittle state after approximately 90 days of degradation correlates with the results presented by Polak-Kraśna et al. [43], who demonstrated that the selective degradation of the amorphous phase, accompanied by a concurrent increase in crystallinity, leads to the loss of plastic deformation capacity, preceding a significant reduction in mechanical strength.

The transient stabilization or even increase in mechanical properties observed around day 60 of degradation is justified by the phenomenon of secondary crystallization. The preferential degradation of amorphous segments promotes the reorganization of shortened polymer chains, resulting in a temporary increase in the degree of crystallinity. Following this stage, a rapid deterioration of mechanical properties occurs, resulting from further depolymerization, chain scission, and the initiation of microcracks [43,48].

The changes recorded in the FTIR spectra are in agreement with the PLLA hydrolysis mechanism described in the literature. The formation of carboxylic groups is accompanied by modifications of the carbonyl band and signals associated with the reorganization of the crystalline structure. The increase in the intensity of $COO^{-}\;$ bands, analyzed using the IDS method, can therefore be considered a reliable indicator of degradation progress [49,50].

In conclusion, the obtained experimental results are consistent with the hydrolytic degradation mechanism of semicrystalline PLLA described in the literature. The characteristic stages of the process—a minor mass loss, a transient stabilization of mechanical properties resulting from secondary crystallization, and the subsequent transition of the material into a brittle state—confirm the typical degradation behavior of this polymer [43,48].

The conducted experimental studies also allowed for the evaluation of the degradation effect on the mechanical properties and structure of the poly(L-lactic acid) (PLLA) scaffolds. Based on these findings, the following conclusions have been formulated:

- The designed geometry of the PLLA scaffolds can be effectively fabricated using the FDM 3D-printing method, ensuring reproducibility and the structural integrity of the obtained specimens.
- During the initial period of incubation in PBS, no significant mass loss of the specimens was detected, indicating the dominance of the material hydration stage and the absence of intensive hydrolytic degradation.
- The progressive degradation of poly(L-lactide) leads to a gradual mass loss of the samples. After 180 days of incubation in PBS solution, the remaining mass was 91.02%, confirming the slow and controlled nature of this process.
- The results of the static tensile test demonstrated that the progressive degradation leads to a decrease in Young’s modulus and tensile strength, with the material exhibiting a transition to a brittle behavior.
- In the static compression test, a relative stability of the mechanical properties was observed up to approximately day 60 of degradation, whereas a distinct deterioration in the material’s mechanical response and a shift in its failure mode were recorded after longer incubation times.
- FTIR spectral analysis confirmed the preservation of the characteristic functional groups of poly(L-lactic acid) and indicated that the dominant degradation mechanism is the hydrolysis of ester bonds, leading to the formation of lactic acid.
- SEM microscopic observations revealed gradual changes in the surface morphology of the scaffolds, manifested by the appearance of irregularities, pits, and structural microcracks, which were particularly prominent after 120 and 180 days of incubation in PBS.
- The obtained results confirm that the degradation of poly(L-lactic acid) affects both the mechanical properties and the surface structure of the scaffolds, which is of crucial importance for their potential application in implantology and tissue engineering.

A notable limitation of this study is the unaddressed impact of FDM process anisotropy—such as raster orientation and interlayer adhesion—on degradation kinetics. Printing parameters define the internal pore architecture, which directly serves as a preferential pathway for water ingress and capillary action, while simultaneously controlling directional mechanical load bearing.

## Figures and Tables

**Table 1:** Comparison of the averaged values of Young’s modulus (E), tensile strength ($R_{m}$), and yield strength ($R_{p0.2}$) obtained from the uniaxial tensile test.

Degradation Time [days]	Young’s Modulus E [MPa]	Tensile Strength $R_{m}$ [MPa]	Yield Strength $R_{p0.2}$ [MPa]
0	2458.07 ± 24.29	45.47 ± 1.3	4.87 ± 0.11
1	1905.37 ± 16.52	55.77 ± 1.1	1.04 ± 0.06
7	1861.21 ± 297.83	38.4 ± 9.63	3.33 ± 1.67
15	2234.59 ± 92.24	43.07 ± 2.93	4.74 ± 0.17
30	2228.19 ± 70.81	41.87 ± 3.64	4.38 ± 0.22
45	2334.49 ± 77.52	44.55 ± 2.41	4.74 ± 0.26
60	2284.74 ± 168.7	36.32 ± 7.95	4.69 ± 0.29
75	2262.28 ± 122.3	37.23 ± 3.3	4.68 ± 0.17
90	2289.9 ± 111.9	36.59 ± 4.11	4.73 ± 0.27
120	2389.21 ± 31.13	46.63 ± 1.32	3.97 ± 0.42
150	2251.24 ± 96.64	41.59 ± 1.69	4.35 ± 0.22
180	2091.48 ± 42.1	31.11 ± 2.97	4.42 ± 0.09

**Table 2:** Comparison of the averaged values of Young’s modulus (E), compressive strength ($R_{c}$), and maximum force ($F_{max}$) obtained from the uniaxial compression test and macro-photographs of PLLA scaffolds obtained after the uniaxial compression test.

Degradation Time [days]	Young’s Modulus E [MPa]	Compressive Strength $R_{e}$ [MPa]	Maximum Force $F_{max}$ [N]	PLLA Scaffold Structure
0	820.81 ± 18.28	47.81 ± 1.21	2659.84 ± 67.6	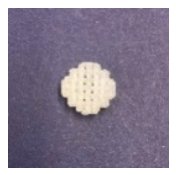
1	567.38 ± 98.95	50.71 ± 0.6	2820.68 ± 33.2	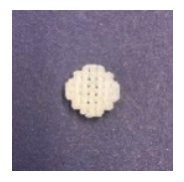
7	583.61 ± 29.31	51.84 ± 0.96	2883.54 ± 53.57	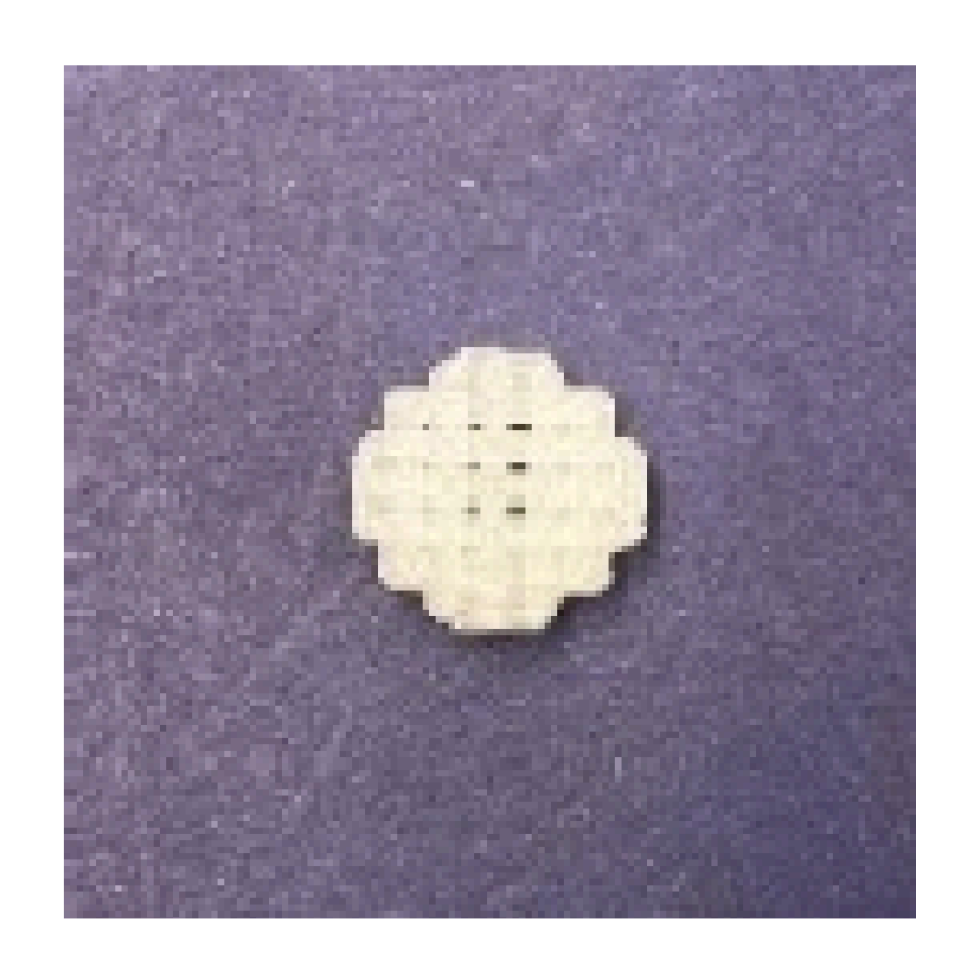
15	675.9 ± 43.98	52.49 ± 1.44	2919.62 ± 80.4	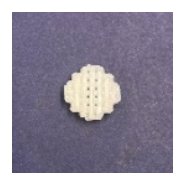
30	619.58 ± 31.98	54.72 ± 1.42	3043.71 ± 78.77	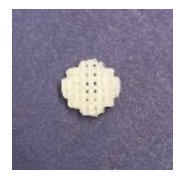
45	710.84 ± 49.27	56.06 ± 0.01	3118.55 ± 0.52	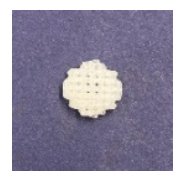
60	650.49 ± 70.66	56.92 ± 0.6	3166.23 ± 33.19	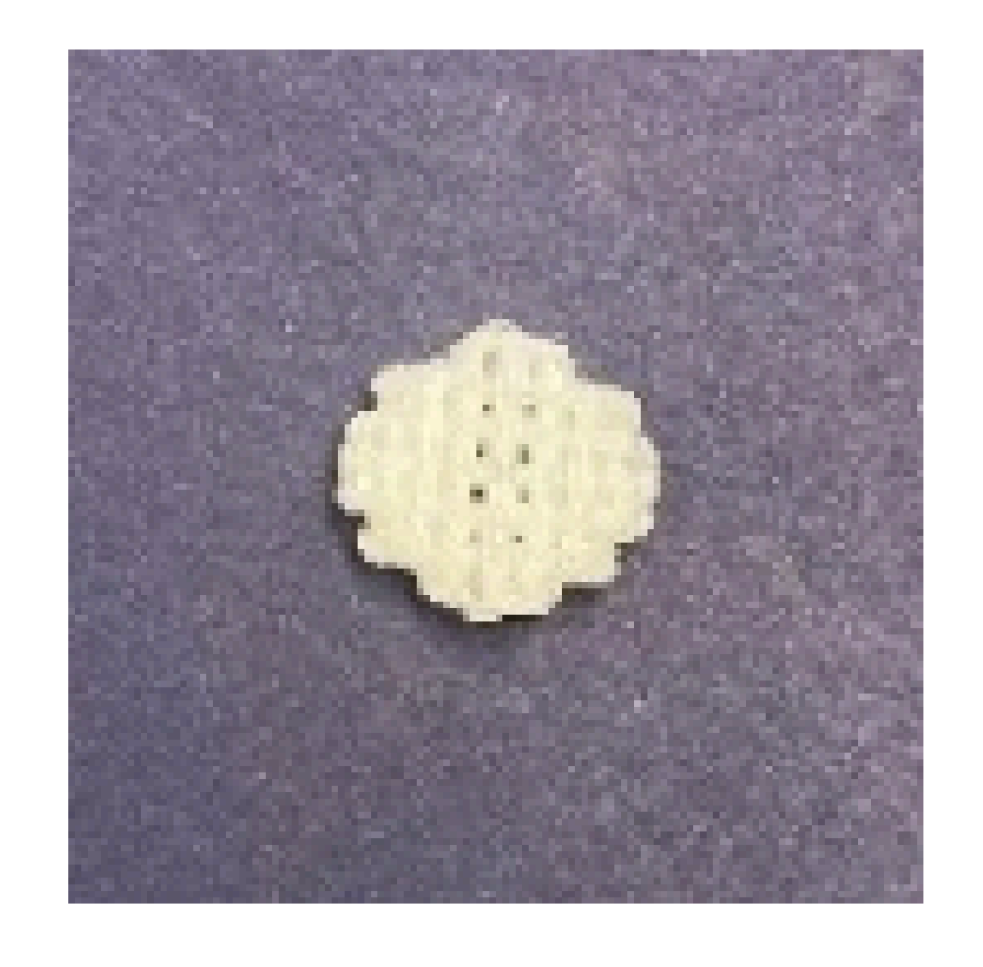
75	667.04 ± 70.28	55.02 ± 0.9	3060.68 ± 49.89	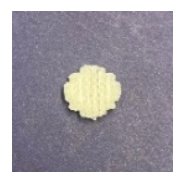
90	482.06 ± 65.94	56.4 ± 0.03	3137.09 ± 1.42	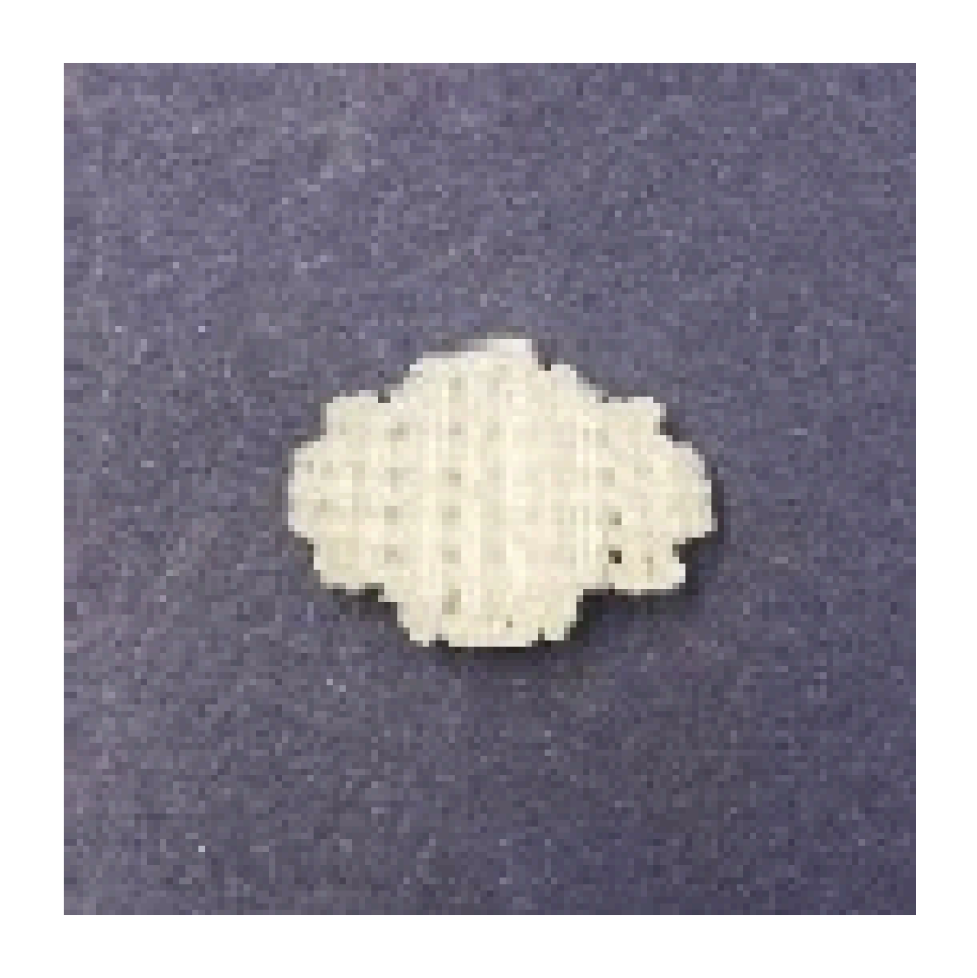
120	615.46 ± 50.17	51.22 ± 3.24	2848.91 ± 180.46	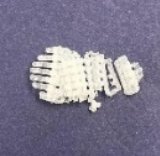
150	695.23 ± 37.69	54.52 ± 0.34	3032.75 ± 18.88	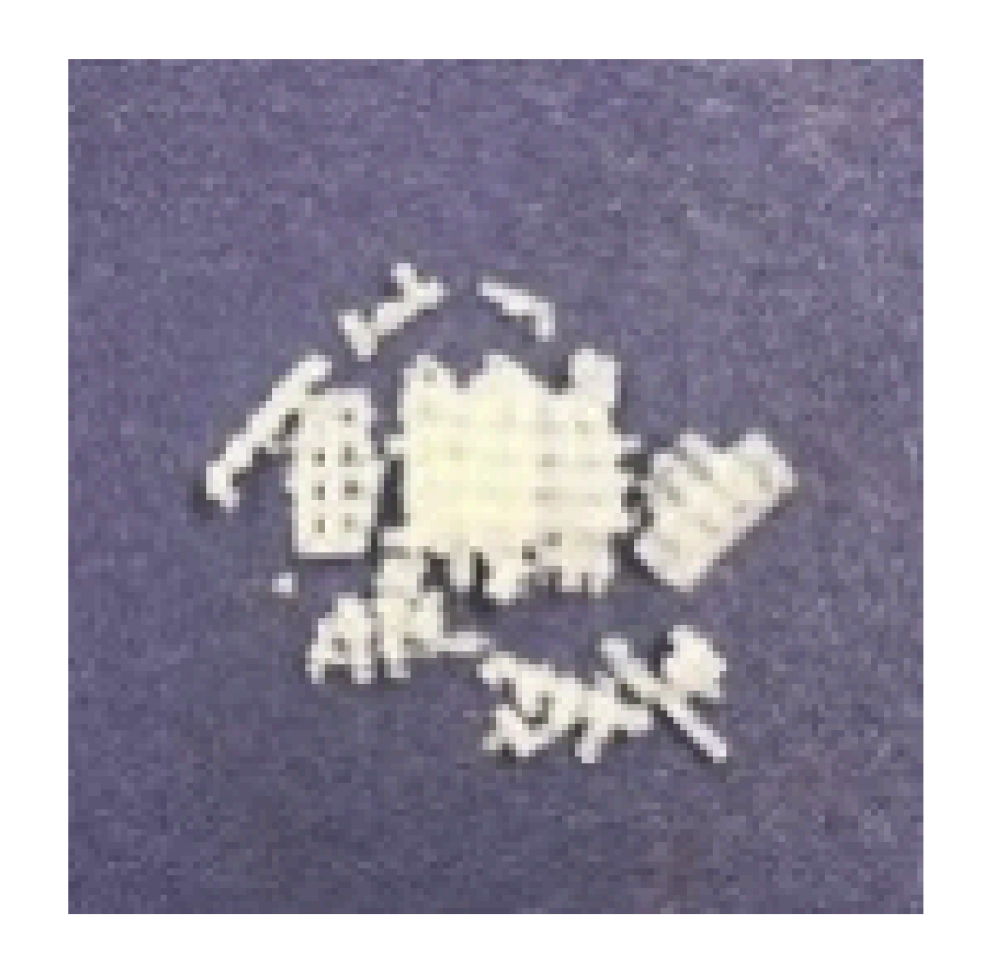
180	727.07 ± 45.05	53.14 ± 0.62	2956.01 ± 34.3	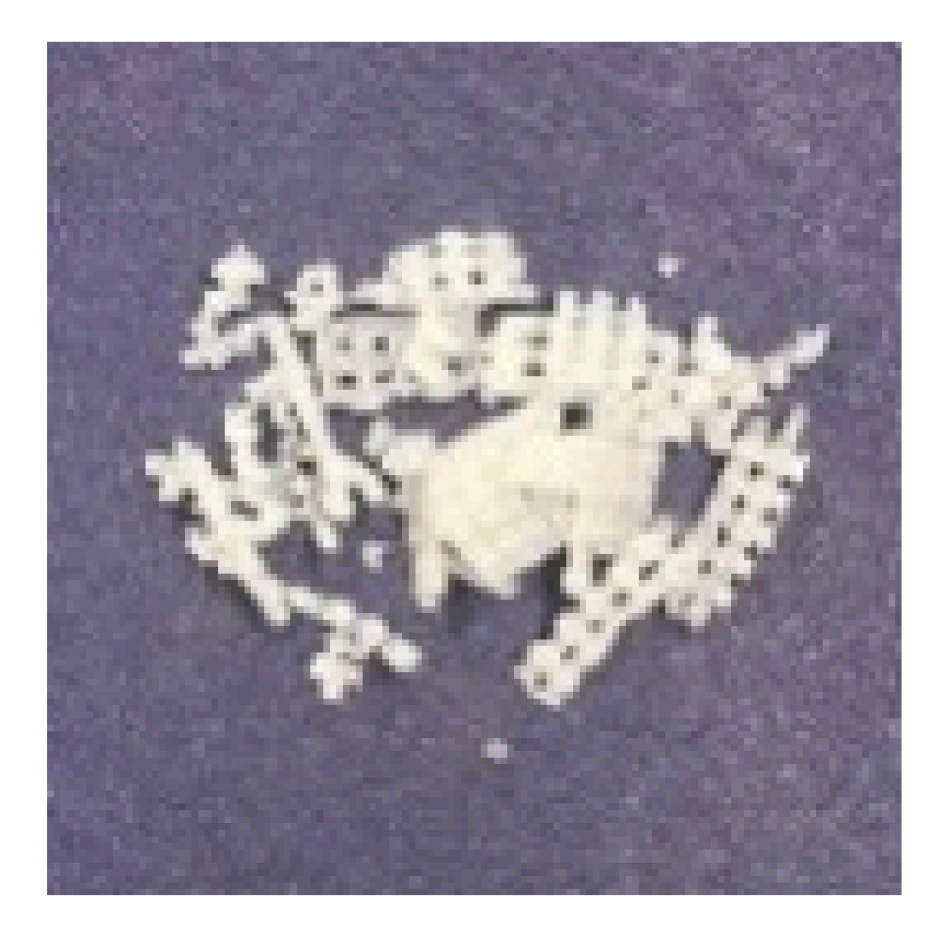

## Data Availability

The original contributions presented in this study are included in the article. Further inquiries can be directed to the corresponding author.
